# Mechanochemical Activation of Mn_3_O_4_: Implications for Lithium Intercalation

**DOI:** 10.1021/acs.inorgchem.4c04660

**Published:** 2025-03-14

**Authors:** Tobias Benjamin Straub, Robert Haberkorn, Guido Kickelbick

**Affiliations:** Inorganic Solid-State Chemistry, Saarland University, Campus, Building C4.1, 66123 Saarbrücken, Germany

## Abstract

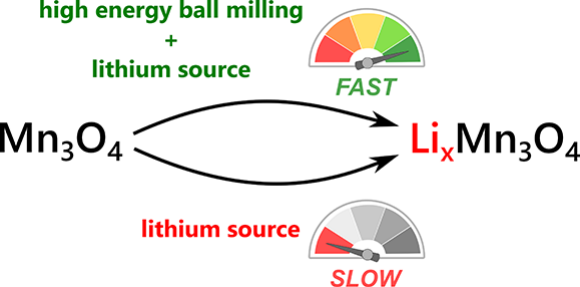

Manganese oxide (Mn_3_O_4_) was subjected to mechanochemical activation (MCA) using a planetary ball mill to investigate the influence of milling parameters on lithium intercalation. After activation, Mn_3_O_4_ was lithiated in suspension with organolithium compounds. Structural changes, including LiMn_3_O_4_ formation, were analyzed by powder X-ray diffraction (PXRD) with Rietveld refinement, supported by scanning electron microscope (SEM), transmission electron microscopy (TEM), physisorption isotherms, and inductively coupled plasma mass spectrometry (ICP-MS). Additional insights into lattice defects were obtained via Raman spectroscopy, electrochemical impedance spectroscopy, and in situ pressure and temperature monitoring during milling. No phase transformation occurred during MCA, though crystallite size decreased to 8.5(5) nm after 4 h at 400 rpm in a zirconia milling jar. Notably, a final crystallite size of 90(9) nm was reached after just 10 min at the same speed. MCA did not cause significant oxygen release from the structure. Short-duration MCA at sufficient speed enhanced lithium intercalation in Mn_3_O_4_, whereas prolonged milling or lower speeds hindered the process. These findings demonstrate that brief mechanochemical activation effectively improves lithium intercalation in transition metal oxides, offering a promising approach for tuning electrochemical properties.

## Abstract 2

High energy ball milling of coarse-grained Mn_3_O_4_, synthesized via a high-temperature solid-state reaction, facilitates rapid lithium intercalation kinetics. The extent of lithiation depends on the lithium source and milling parameters employed.

## Introduction

Manganese(II,III) oxide (Mn_3_O_4_) is a redox active transition metal oxide with versatile applications ranging from supercapacitors^1^ and catalytic oxidation of air pollutants^2^ to improving hydrogen storage in magnesium hydride systems.^3^ The high theoretical capacity (936 mA h g^–1^) makes it an attractive candidate for lithium-ion batteries and other energy storage applications, such as supercapacitors, positioning Mn_3_O_4_ as a promising material for next-generation battery technologies.^4,5^

Occurring mainly in crystalline form, trimanganese tetraoxide exhibits a spinel structure (space group *I*4_1_/*amd*) in which Mn^2+^ ions occupy one eights of the tetrahedral sites and Mn^3+^ ions occupy half of the octahedral sites within a cubic close-packed lattice of O^2−^ ions. The Jahn–Teller effect in combination with Mn^3+^ (d^4^) leads to a tetragonally distorted spinel structure.^6,7^ Three polymorphs of Mn_3_O_4_ are described in the literature, with tetragonal α-Mn_3_O_4_ transforming into cubic β-Mn_3_O_4_ at around 1450 K, while a metastable phase (γ-Mn_3_O_4_) can be synthesized under high temperature and pressure conditions.^8−11^ In rechargeable lithium-ion batteries, α-Mn_3_O_4_ is commonly used in various geometric forms such as ordered aligned nanostructures, nanorods or nanosized sponge-like Mn_3_O_4_ as anodes.^5,12−14^ The crystal structure of LiMn_3_O_4_ is closely related to that of Mn_3_O_4_, as it has the same space group and also shows a tetragonal distortion. In LiMn_3_O_4_ both manganese and lithium atoms occupy all the octahedral sites formed by the oxygen sublattice, similar to the NaCl-type structure.^15^

The synthesis of Mn_3_O_4_ has been extensively investigated in the literature using various methods. These include high-temperature synthesis from MnCO_3_^16^ or MnO_2_,^12^ solvothermal synthesis,^17^ gas–liquid one-pot synthesis,^18^ decomposition of metal organic precursors,^3^ and electrochemical methods.^5^ Many of these methods are aimed at the production of defect-free, thermodynamically stable products. In our recent work, we presented a new approach for Mn_3_O_4_ synthesis: a solvent-free mechanochemical synthesis in which stoichiometric amounts of MnO and Mn_2_O_3_ are reacted at room temperature by high-energy ball milling (hebm).^19^ This method led to the production of Mn_3_O_4_ with the smallest crystallite size ever reported (14.2 nm). Our first investigations have shown that the obtained tetragonal α-Mn_3_O_4_ exhibits accelerated lithiation compared to materials derived from high-temperature syntheses. In addition, mechanochemical activation has shown remarkable potential to improve the lithium ion conductivity of various systems, including titanates.^20^

High-energy ball milling is a powerful technique for the treatment of solids that offers a variety of benefits, such as particle size reduction, the generation of reactive surfaces and the conversion of reactants.^21,22^ Further advantages of mechanochemistry are the absence of solvents,^23^ the minimization of byproducts,^19^ the lower reaction temperatures,^22^ shorter reaction times,^24^ and the formation of metastable phases often accompanied by an increased defect formation.^25^ This mechanochemical activation can increase the chemical activity and induce structural disorder and amorphization of the material.^26^ Studies in the literature have emphasized the remarkable properties of manganese oxides produced by mechanochemical activation, including enhanced oxygen exchange and accelerated redox processes.^27,28^ Consequently, this activation method is promising to increase the surface reactivity and catalytic activity of manganese oxides.^29^ In addition, the reduction in crystallite size induced by milling leads to significant changes in lattice parameters, which can be attributed to the Jahn–Teller effect.^28^ Another remarkable phenomenon observed during mechanochemical treatments is the formation of oxygen defects within the crystal lattice, which is accompanied by the release of oxygen. This effect has been documented in various materials such as MnO_2_, LiNbO_3_ or ZnFe_2_O_4_.^30−32^

An attractive method for detecting changes in the reactivity of mechanochemically activated potential intercalation host materials is their chemical lithiation in suspension after MCA. Organolithium compounds commonly used for such reactions include *n*-butyllithium, *tert*-butyllithium, or lithium naphtalene.^33^ Despite the importance of this method for the investigation of electroless lithiation of potential electroactive oxides, the chemical lithiation of manganese oxide compounds has only been investigated in a few studies to date. Farcy et al. showed that lithium insertion into cation-deficient mixed Mn–Co spinel oxides does not reveal any change in the original structure, but the cation vacancies hinder obviously the Li-cation transport.^34^ In addition, Dose, Lehr and Donne simulated the discharge behavior of a battery cathode by chemical lithiation of MnO_2_ and analyzed the changes in crystal structure, chemical composition and morphology. Their results indicate that lithium intercalation leads to a phase transition to LiMn_2_O_4_, which is further reduced to Li_2_Mn_2_O_4_, accompanied by an initial decrease in Brunauer–Emmett–Teller (BET) surface area.^35^

In the present study, we systematically investigate the differences in the lithiation behavior between high-temperature synthesized Mn_3_O_4_ and mechanochemically activated samples. We hypothesize that the intercalation tendency is closely related to crystallite size and defect concentration, both of which are modulated by the mechanochemical treatment. The effects of milling parameters and variations in lithiation reactions were methodically investigated in order to elucidate the mechanisms underlying these phenomena.

## Experimental Section

### Materials

*n*-Butyllithium (2.5 M in *n-*hexane, Acros, Geel, Belgium), *tert*-butyllithium (1.7 M in *n*-pentane, Sigma-Aldrich, Steinheim, Germany), *n*-hexyllithium (2.3 M in *n-*hexane, Sigma-Aldrich), methyllithium (1.6 M in diethyl ether, Sigma-Aldrich, Steinheim, Germany), tetrahydrofuran (99.9+%, Th. Geyer, Renningen, Germany), 2-propanol (97%, Biesterfeld Spezialchemie, Hamburg, Germany), *n*-hexane (97%, VWR, Darmstadt, Germany), *n*-pentane (99%, Stockmeier Chemie, Bielefeld, Germany), acetonitrile (>99.9%, Th. Geyer, Renningen, Germany), diethyl ether (>99.7%, Fisher Scientific, Dreieich, Germany), MnCO_3_ (99.9+%, Sigma-Aldrich, Steinheim, Germany), Li_2_S (98%, abcr, Karlsruhe, Germany), and diphenylacetic acid (99%, Sigma-Aldrich, Steinheim, Germany). All received powdery reactants were characterized by X-ray powder diffraction before usage. Due to the rapid deterioration of organolithium reagents their concentration was regularly determined by titration against diphenylacetic acid.^36^ Solvents were purified and dried using a Solvent Purification System (MBRAUN, Garching, Germany) and stored under argon and over molecular sieves. All chemicals were used as received without further purification.

### Oven-Equipment

Muffle furnace N11/HR (Nabertherm, Lilienthal, Germany) with control unit C30 and a maximum temperature of 1280 °C.

### Characterization

Powder X-ray diffraction (PXRD) patterns of the pulverized samples were recorded at room temperature on a D8-A25-Advance diffractometer (Bruker, Karlsruhe, Germany) in Bragg–Brentano θ–θ-geometry (goniometer radius 280 mm) with Cu *K*_α_-radiation (λ = 154.0596 pm). A 12 μm Ni foil working as *K*_β_ filter and a variable divergence slit were mounted at the primary beam side. A LYNXEYE detector with 192 channels was used at the secondary beam side. Experiments were carried out in a 2θ range of 7–120° with a step size of 0.013° and a total scan time of 2 h. The background caused by white radiation and sample fluorescence was reduced by limiting the energy range of the detection. The recorded data were analyzed with the Bruker TOPAS 5.0 software using Rietveld refinement.^37^ The mean crystallite size was calculated as the mean volume weighted column height derived from the integral breadth (L_Vol_(IB)). Instrumental broadening and strain were taken into account by using the fundamental parameters approach, as it is implemented in the program TOPAS.^38^ The instrumental broadening was empirically determined by a set of four reference materials. The required structure files were obtained from the relevant literature or the Inorganic Crystal Structure Database (ICSD). Modeling strain within the program TOPAS is limited to a symmetrical broadening of the reflections. Lattice parameter variations due to the distribution width of the content of Li after lithiation provide also peak broadening by strain. In order to enable a more complex model of strain a multifraction model was used for a more proper description of the diffraction patterns. For each phase used, up to three fractions with slightly different lattice parameters were used.^39−42^ The other parameters were kept the same for each fraction.^43^ Within the Supporting Information a parameter *q* is defined, enabling a quantification of the tetragonal distortion.

The elemental composition of the samples was determined using inductively coupled plasma mass spectrometry (ICP-MS) with a 8900 Triple Quad ICP-MS system equipped with a SPS4 autosampler (Agilent, Santa Clara, USA). Stock solutions of single element ICP-MS standards of Li^+^ (Merck Millipore, Darmstadt, Germany), Mn^2+^ (Sigma-Aldrich, Steinheim, Germany) and Sc^3+^ (Merck Millipore, Darmstadt, Germany) were used. The detector dwell time was 100 μs, the repetition was 3 times, and the measured isotopes were ^7^Li in no gas mode and ^55^Mn using He as collision gas as well as ^45^Sc (all used modes) as internal standard.

Transmission electron micrographs were recorded on a JEM-2010 electron microscope (JEOL, Tokyo, Japan) with 200 kV used for electron acceleration. Samples were prepared by drop coating nanoparticle dispersions in 2-propanol on Plano S160–3 carbon coated copper grids.

For the acquisition of SEM images, the sample was mounted on a carbon adhesive film and then sputtered with a thin layer of gold. The images were taken with the microscope model JSM-7000 F (JEOL, Tokyo, Japan), the working distance was 10 mm, and a voltage of 20 kV was applied.

The nitrogen sorption analyses at −196 °C were carried out using a Quadrasorb IQ system (Anton Paar, Graz, Austria). Before each measurement, the materials were outgassed for 12 h at 100 °C under vacuum. A two-dimensional (2D) nonlocal density functional theory approach was applied to determine the cumulative specific surface area.

The recording of the Raman spectra was performed on a Raman microscope spectrometer, LabRAM HR Evolution (HORIBA Jobin Yvon, Longmujeau, France) equipped with a 633 nm He–Ne Laser (Melles Griot, IDEX Optics and Photonics, Albuquerque) and a 600 lines/mm grating was used.

The measurements for electrochemical impedance spectroscopy were carried out at ambient temperature using an MTZ-35 impedance analyzer (BioLogic, Seyssinet-Pariset, France) with a frequency range of typically 100 mHz to 100 kHz. The powdery samples were pressed to pellets of a diameter of about 7 mm and a thickness of 1–2 mm by uniaxial pressing (2 t load) followed by isostatic pressing (300 kN). The top and bottom of the pellets were coated with silver conductive paste to establish the electrical contact to the electrodes of the spectrometer. Due to limitations of the measurement equipment and limited quality of the coating for some samples, data at the lowest or highest frequencies could not be used for evaluation. The program electrochemical impedance spectroscopy (EIS) Spectrum Analyzer 1.0 was used for fitting of Nyquist-plots.^44^ The other steps of evaluation of the impedance spectra were done by programs of our own. The parameter σ_0_ of the conductivity was approximated by the mean value of σ′ of the three lowest evaluable frequencies. The parameters *s* and *p* were estimated by graphically supported simulations.

### Synthesis

#### Solid-State Synthesis

In the high-temperature synthesis of Mn_3_O_4_, approximately 15.0 g of manganese carbonate (MnCO_3_) was placed in a platinum crucible, heated to 1000 °C in a muffle furnace at a heating rate of 250 °C per hour, and held for 10 h at the final temperature. The red-hot crucible was then transferred to an argon-flushed desiccator for rapid cooling, preferably without contact with oxygen. The desiccator was quickly closed and evacuated. The Mn_3_O_4_ obtained in this way is a reddish-brown solid.^16,19^

#### Milling of Mn_3_O_4_

In this study, Mn_3_O_4_ was activated by hebm for different periods of time before lithiation. For this purpose, 3.0 g of Mn_3_O_4_ was placed in a 45 mL grinding jar filled with 180 grinding balls (diameter: 5 mm). The grinding jar and balls were made of yttria-stabilized zirconia. The rotational speeds were 200, 400, and 600 rpm for different periods of time (no hebm, 10 min, 30 min, 1, 2, 4, and 8 h) in a PULVERISETTE 7 *premium line* planetary ball mill (Fritsch, Idar-Oberstein, Germany). The ratio of grinding balls to powder was 23 to 1 and 200 μL 2-propanol were added as a dispersion agent to prevent cementation of the powder.

#### Lithiation

1.0 g of Mn_3_O_4_ (pristine or milled) was placed in a dry three-necked flask, then 25 mL of absolute solvent and 2.5 equiv (except for Li_2_S, here 5 equiv were used^45^) of the appropriate organolithium reagent were added and afterwards stirred for different times (3, 6, 18, 24, 48, and 96 h) under argon at room temperature. The resulting solid was filtered off under argon atmosphere through a frit, first washed with the corresponding absolute solvent and afterward with 2-propanol, to remove and quench any remaining organolithium reagent. Residual solvent was removed by vacuum and the solid obtained was subsequently characterized. Upon lithiation, the solid undergoes a color transition from reddish-brown to dark gray/black.

## Results and Discussion

### Synthesis of Mn_3_O_4_ Applying a High-Temperature Method

The starting material (Mn_3_O_4_) was produced by a thermal decomposition of MnCO_3_ at 1000 °C in a muffle furnace according to literature procedures.^16,19^

The characterization of the pristine product, obtained by high-temperature synthesis and of the milled samples was performed by PXRD. Rietveld refinement shows that coarse-grained, phase-pure Mn_3_O_4_ without any visible impurities or byproducts, with a crystallite size larger than 500 nm was obtained (Figure S1). The analysis of the resulting sample reveals a lattice parameter *a* of 576.29(1) pm and 946.80(1) pm for *c* in the body-centered tetragonal unit cell, which agrees well with previously published data.^14,19,46,47^ The sample used as a reference material for pristine Mn_3_O_4_ provides a distortion *q* = 16.17%. Tables S1 and S2 in the Supporting Information show further data of Rietveld refinement and the definitions of the different *R*-factors.

The coarse-grained Mn_3_O_4_ produced via high-temperature synthesis served as the starting material for further experiments.

### Mechanochemical Activation of Mn_3_O_4_

The MCA parameters for Mn_3_O_4_ were optimized to maximize impact while minimizing abrasion by considering the jar material, ball count, and rotational speed. To achieve better fits for the mechanochemically activated Mn_3_O_4_ and its lithiation product Li_*x*_Mn_3_O_4_ (0 ≤ *x* ≤ 1) in the Rietveld refinement, a multifraction model was used to describe each phase. Further details on the model and an exemplary refinement using three fractions are provided in the SI (Figure S2).

**Figure 1:**
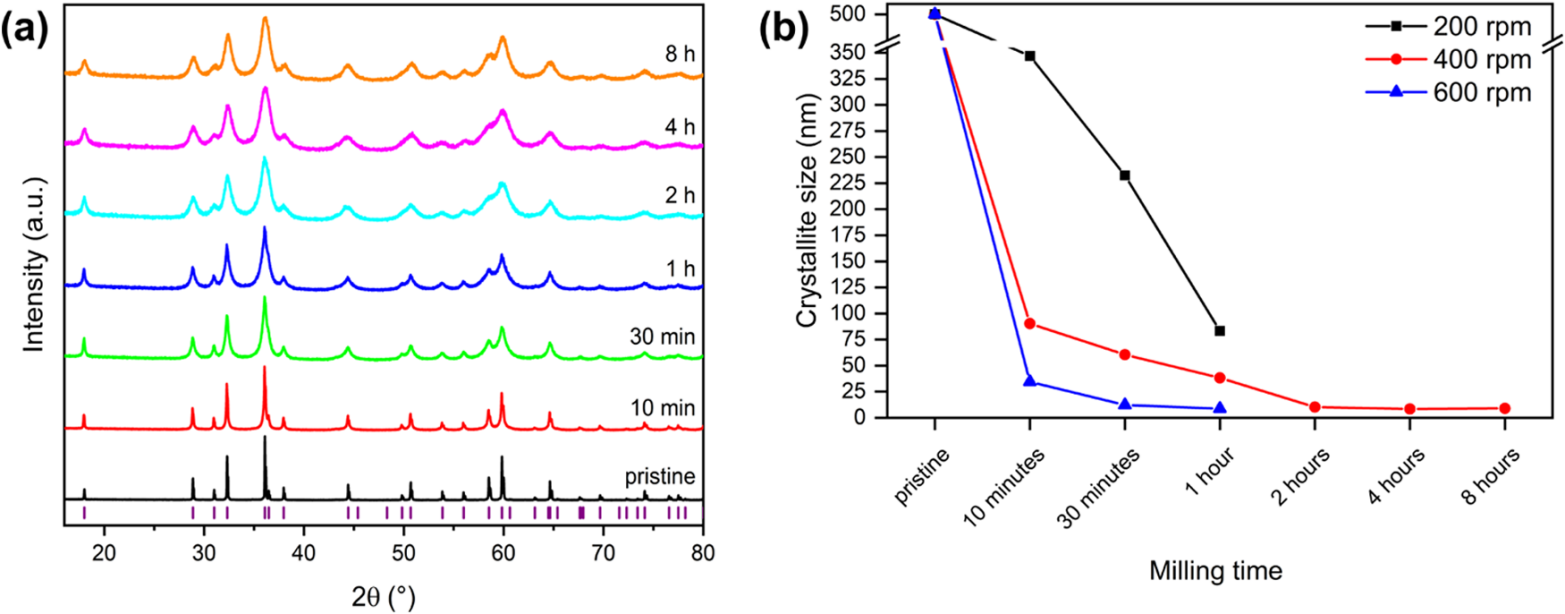
Enlarged section of the PXRD patterns of Mn_3_O_4_ milled at 400 rpm for different times (a) and the evolution of the crystallite size at different rotational speeds and different time lengths (b). The purple lines indicate the *hkl*s of Mn_3_O_4_. Crystallite size was determined by Rietveld refinement proportionally weighted from the multifraction model. The lines in the figure connecting the measured points are only orientation aids for the eye.

After milling, the diffractograms of the mechanochemically activated Mn_3_O_4_ do not show any phase transitions. But a broadening of the reflections due to the reduction of the crystallite size during the grinding process can be observed and also by strain parametrized by a conventional strain model and–if necessary–an additional multifraction model. Discrete values of strain were determined using models with a single fraction per phase. Furthermore, we also cannot detect an amorphization of the sample applying the chosen milling conditions. The comparison of the integral area under the reflections shows no significant differences when comparing pristine and mechanochemically activated Mn_3_O_4_, which means there is no loss of integral intensity during grinding (Figure 1a).

Mechanochemical activation can have a huge impact on the crystallite size, which also influences diffusion phenomena in solid-state chemistry and therefore lithiation processes. The crystallite size of Mn_3_O_4_ continuously decreases for grinding times of up to 2 h at constant rotational speed of 400 rpm (Figure 1b). After 2 h, a reduction of the initial size from over 500 nm to 10.2(6) nm is achieved. Longer grinding times result in only very small reductions in crystallite size. The development of the crystallite size was also analyzed for shorter grinding times (up to 1 h) at two other speeds, namely 200 and 600 rpm. While at 200 rpm the determined sizes are higher than at the other two speeds at identical time intervals, at 600 rpm a much faster reduction of the crystallite size can be observed. Under these conditions a value of 9(3) nm is already achieved after 1 h (Table S3).

The smallest crystallite size under the given grinding conditions is 8.5(5) nm, which is again much smaller than the crystallite size of 14.2(2) nm obtained by Becker in the mechanochemical synthesis of Mn_3_O_4_.^19^Figure S3 in the SI depicts the variation of PXRD patterns at different rotational speeds and time periods.

**Figure 2:**
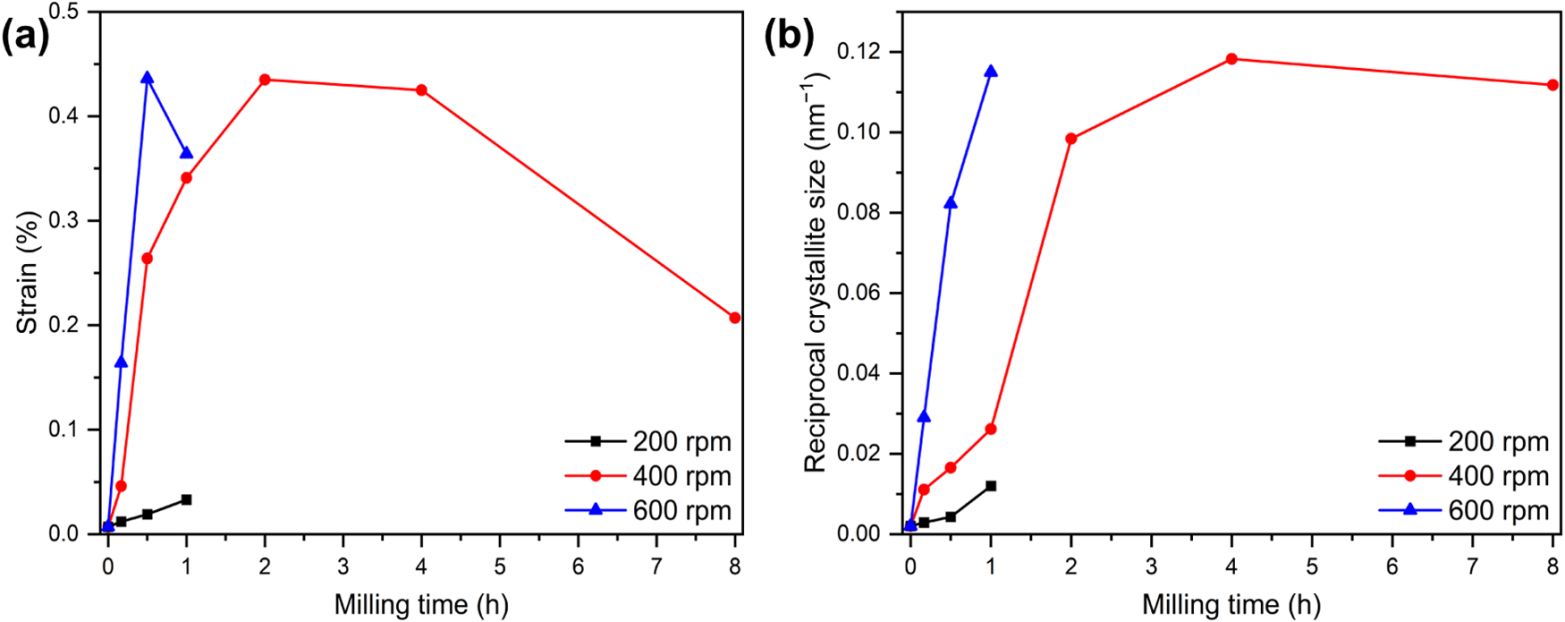
(a) Strain and (b) reciprocal crystallite size of Mn_3_O_4_, milled at different rotational speeds and different periods. The lines in the figure connecting the measured points are only orientation aids for the eye.

Another crucial parameter to consider is microstrain, which arises predominantly due to high-energy ball milling. The calculated strain is highly dependent on both the milling duration and the rotational speed (Figure 2a). At higher rotational speeds, strain increases significantly, particularly within the first hour of milling. However, after reaching a maximum value of approximately 0.45%, it begins to decrease with further mechanical loading. This peak strain is observed after around 4 h at 400 rpm, whereas at 600 rpm, it occurs within just half an hour. This trend is further corroborated by an analysis of the reciprocal crystallite size, which serves as an indicator of reflex broadening. The broadening effect intensifies with increasing rotational speed but gradually approaches a saturation point over prolonged milling times (Figure 2b). Notably, at higher rotational speeds, the broadening effect develops more rapidly.

Strain is the result of different types of stress. While microstrain (i.e., caused by point defects) has a short-range other types of stress, such as dislocations, stacking faults or microtwinning, can have a long-range. Very small crystallites, as in our case, tend to avoid stresses with a long-range, while point defects can survive even in very small nanocrystals.^48,49^ A crystallite size smaller than approximately 25 nm seems to exhibit less long-range stress compared to larger crystallites.

**Figure 3:**
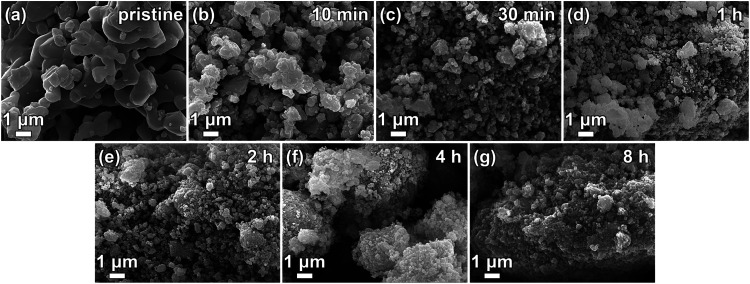
SEM images of pristine (a) and milled Mn_3_O_4_ at different milling times with constant rotational speed of 400 rpm, 10 min (b), 30 min (c), 1 h (d), 2 h (e), 4 h (f) and 8 h (g). Factor of magnification 10,000.

The scanning electron microscope (SEM) images of the ground Mn_3_O_4_ at different time periods show that the size and morphology of the crystals change with increasing grinding time (Figures 3 and S4). The pristine starting material shows micrometer-sized intergrown crystals with smooth edges. With continuous milling time the size of the crystals decreases, the edges get a sharper profile, and the particles tend to agglomerate to larger clusters. A higher rotational speed with the same milling time leads to a faster decrease of the crystal size. SEM analysis was also supported for selected samples by transmission electron microscopy (TEM) studies (Figure S5). The TEM images do not indicate any amorphous phase around or between the particles.

Nitrogen physisorption measurements were used to determine the development of the specific surface area (Figure S6) and the cumulative pore volume (Figures S7 and S8) at different milling times as a function of the rotational speed more precisely at 200, 400, and 600 rpm. The analysis shows that the samples have a very small surface area and are therefore subject to error and not suitable for making a reliable statement, but they are suitable for showing a general trend. The pristine starting material has a specific surface area of 1.4 m^2^/g. After a milling time of 10 min at 400 rpm, an increase in the specific surface area can be observed up to 16.6 m^2^/g. After 30 min at 400 rpm, a local minimum of specific surface area was observed (6.4 m^2^/g), and from then on, a steady increase in surface area was observed at longer grinding times. For example, 18.0 m^2^/g after 2 h and the highest value of 39.6 m^2^/g at the longest grinding time (8 h). The individual measurement values are listed in Table S4. Corresponding nitrogen physisorption data are presented in the SI (Figures S9 and S10).

The models for the development of the specific surface area in MCA comprise three stages: First, the surface area increases due to the fragmentation of the particles in relation to the grinding time or energy input, then the increase slows down because the particles start to aggregate, and finally the specific surface area decreases due to the formation of agglomerates or amorphization.^26,50,51^ The XRD and TEM images give no indication of amorphization of the samples, but the formation of agglomerates/aggregates can be observed in the SEM images. Several literature examples exhibit a similar trend in specific surface area during grinding, with a local minimum. This behavior can be attributed to either a phase transformation after prolonged grinding or a mechanochemical reaction.^52−54^ No phase transformation or mechanochemical reaction evidence was observed in the XRD patterns. Therefore, another reason must account for the surface area increase. The cumulative pore volume increased with longer grinding times indicating additional pores were created and/or became accessible due to grinding. This may result from agglomerate formation, leading to interparticle/extrinsic voids.^55−57^ The effects are discussed in the section examining MCA’s influence on lithiation.

Abrasion and thus contamination of the samples can be a problem in mechanochemical studies. Therefore, we investigated the effects of different grinding times on the abrasion of yttrium-stabilized zirconia grinding balls and jars using ICP-MS analysis. Yttrium content remained below the detection limit, while zirconium was detected only after prolonged milling (>2 h). The zirconium content in the product was 0.02 wt % after 2 h, 0.03 wt % after 4 h, and 0.04 wt % after 8 h of grinding.

**Figure 4:**
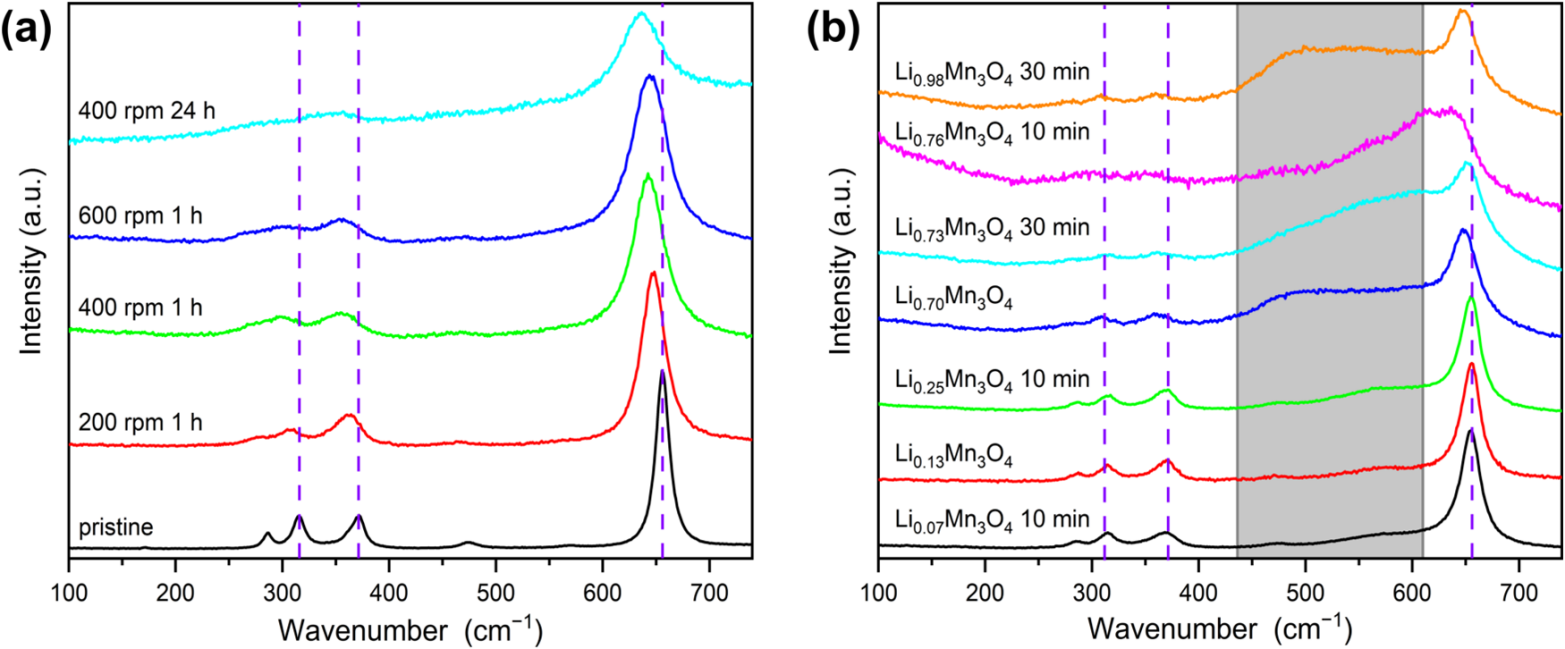
Enlarged section of the Raman spectra (633 nm) of pristine and milled Mn_3_O_4_ under different conditions (a) and of Li_*x*_Mn_3_O_4_ (b) with different lithium contents, partly additionally milled for different times at 400 rpm. The dashed purple lines are used to illustrate the shift of the peaks, and the gray area shows the appearance of new peaks.

Raman spectra were recorded from both pristine and milled Mn_3_O_4_ samples. All spectra show the characteristic peaks of Mn_3_O_4_ in the depicted range, no additional peaks are detected, suggesting the absence of additional phases or transformations induced by grinding. However, comparison of the spectra showed a broadening of the peak corresponding to the Mn–O stretching vibrations (A_1g_ symmetry mode) of Mn^2+^ in tetrahedral environments at 654 cm^–1^ is observed for the milled samples (Figure 4a).^58,59^ The broadening of the peak is more pronounced the harsher the grinding conditions are. In addition, the three less intense peaks between 275 and 375 cm^–1^ also exhibited broadening, with longer grinding times causing them to merge with the baseline. A blueshift to lower wavenumbers can also be observed (dashed, purple lines). This can be explained by the phonon confinement effect, which is based on the reduction in grain size and not on oxygen defects. In this case, a red shift should be observed.^60,61^ The increasing broadening of the Raman peaks (full width at half-maximum) is an indicator of increasing structural disorder in a crystalline system, because it is known that hebm can lead not only to a reduction in particle size, but also to the appearance of defects in the form of structural disorder, the formation of vacancies, lattice strains and deformations.^62−66^ The lithiated Mn_3_O_4_ samples also show broadening of the peaks and a blue shift of the peaks belonging to Mn_3_O_4_ (Figure 4b). In addition, a new broad peak has formed in the gray area, which is probably due to the intercalation of lithium.

To investigate whether grinding Mn_3_O_4_ leads to significant oxygen vacancy formation by oxygen gas release, as observed for other oxides, an experiment was conducted using a grinding jar with a pressure and temperature sensor lid.^30−32^ In contrast to observations with other oxide materials, our results show no significant oxygen release under the applied grinding conditions. The slight pressure increase can be attributed to the 14 K temperature rise in the grinding jar due to the grinding process (Figure S11). This suggests a distinctive behavior of Mn_3_O_4_ compared to other binary or ternary oxides, where oxygen vacancies are known to form during ball milling. Similarly, the Raman spectra show no evidence of the formation of oxygen vacancies (shift to higher wavenumbers).

### Lithiation of Mn_3_O_4_

**Scheme 1:**
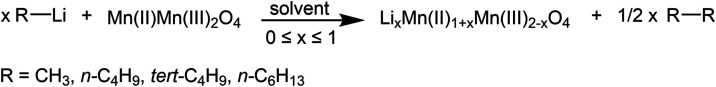
General Reaction Equation for the Lithiation of Mn_3_O_4_ with Organolithium Compounds The reactions were carried out in suspension in the solvents *n*-hexane, THF or diethyl ether.

Scheme 1 shows the general equation of lithiation of Mn_3_O_4_ with various organolithium compounds. Beside *n*-butyllithium we also used *tert*-butyllithium, *n*-hexyllithium and methyllithium as lithiation reagents in our experiments. A multiphase model was also employed to describe the lithiation process of Mn_3_O_4_ in the Rietveld refinement. An intermediate tetragonal transition state was identified, with additional details provided in the SI (Figures S12, S13 and Table S5).

**Figure 5:**
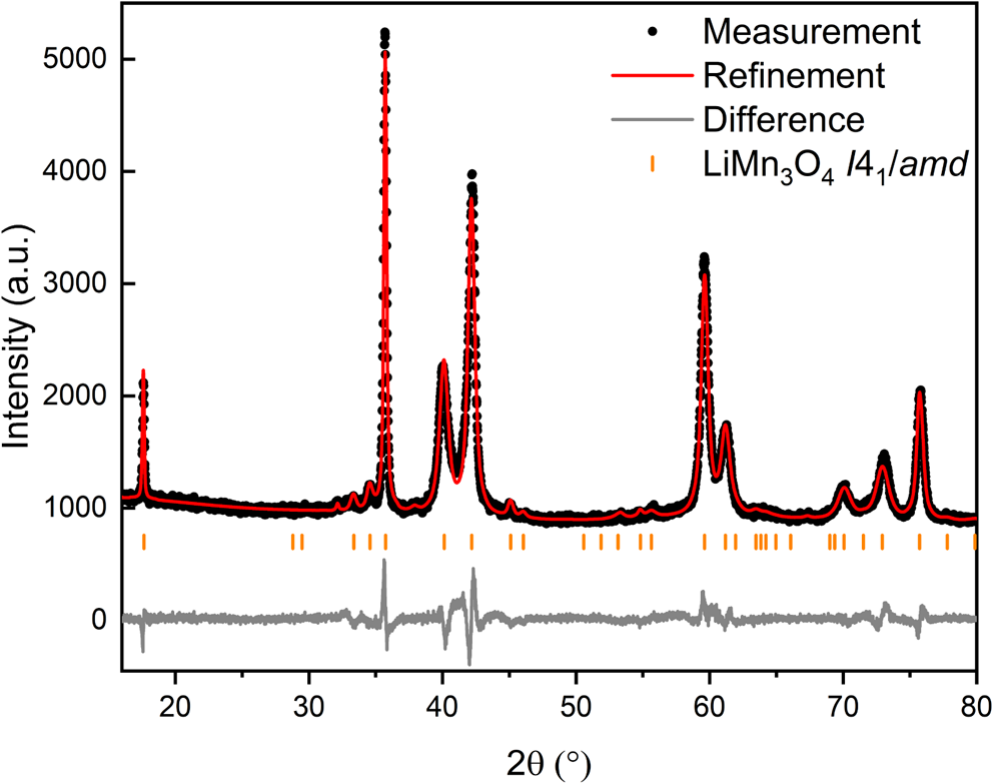
Enlarged section of the Rietveld plot of LiMn_3_O_4_ obtained via lithiation of Mn_3_O_4_ with an excess of *n*-butyllithium in *n*-hexane. LiMn_3_O_4_ in SG *I*4_1_/*amd* with *a* = 605.39(2) pm, *c* = 898.12(5) pm and *V* = 0.32915(3) nm^3^. Refinement parameters: number of independent parameters = 30, *R*_wp_ = 8.06%, *R*_exp_ = 5.16%, GOF = 1.56.

We first investigated the maximum lithium intercalation of Mn_3_O_4_ by reacting activated Mn_3_O_4_ (10 min, 200 rpm) with an excess of *n*-butyllithium in *n*-hexane under reflux. First, 2 equiv of *n*-butyllithium were used for 2 days, followed by another 2 days with 5 equiv. This sequential lithiation process yielded LiMn_3_O_4_ as the final product, with no further increase achievable. PXRD analysis and Rietveld refinement confirmed the recovery of phase-pure LiMn_3_O_4_ (Figure 5). The Rietveld refinement analysis reveals that LiMn_3_O_4_ has lattice parameters *a* = 605.39(2) and *c* = 898.12(5) pm, with a corresponding cell volume of 0.32915(3) nm^3^ and a tetragonal distortion *q* of 4.9%. Further refinement data for LiMn_3_O_4_ are listed in Tables S1 and S2. This sample exhibited the highest unit cell volume among all samples and was used as a reference, assuming a molecular formula Li_1.00_Mn_3_O_4_. The determined unit cell volume exceeds the values reported in the literature. Goodenough and Thackeray reported a cell volume of 0.32678 nm^3^ and Becker reported 0.3261(2) nm^3^, all significantly lower than our results.^15,19,67^ In addition the tetragonal distortion *q* is smaller than the values derived from lattice parameters described in literature. The tetragonal distortion is caused by the Jahn–Teller-effect of Mn(III) residing in octahedral sites. LiMn(II)_2_Mn(III)O_4_ has a lower concentration of Mn(III) than Mn(II)Mn(III)_2_O_4_ and therefore a lower tetragonal distortion. The higher the value of *q* is, the more Mn(III) and the less Li will be found within the phase Li_*x*_Mn(II)_1+*x*_Mn(III)_2–*x*_O_4_.

This discrepancy suggests that the samples described in the literature may not have been as fully lithiated as our reference sample. A possible explanation could be that neither Becker nor Goodenough and Thackeray performed a second lithiation step with a large excess of *n*-butyllithium on the already lithiated product. Efforts to gain further insight into the intercalated lithium using solid-state NMR spectroscopy were hampered by the paramagnetic behavior of the substance, which makes NMR measurements infeasible. Even attempts to alleviate this problem by diluting the sample with NaCl were unsuccessful.

Table 1 summarizes key parameters of individual phases, including lattice parameters, cell volume and tetragonal distortion *q*, for Mn_3_O_4_ and for its lithiation product Li_*x*_Mn_3_O_4_. Literature data and experimentally determined values from our samples are presented.

**Table 1:** Lattice Parameters *a* and *c*, Cell Volume *V* and Tetragonal Distortion *q* of Some Samples of Mn_3_O_4_ and Li_*x*_Mn_3_O_4_

phase	*a*/pm	*c*/pm	*V*/nm^3^	*q*/%	source
Mn_3_O_4_	576.29	946.80	0.31444	16.2	this work
	576.3	945.6	0.31405	16.1	Boucher^46^
Li_∼0.4_Mn_3_O_4_	592.66	911.55	0.32018	8.8	this work
LiMn_3_O_4_	605.39	898.12	0.32915	4.9	this work
	602.2	901.1	0.32678	5.8	Goodenough^15^
	601.9	900.1	0.32609	5.7	Becker^19^

In order to clarify the influence of mechanochemical activation on the lithiation behavior of Mn_3_O_4_, we conducted a kinetic lithiation study. In this study, different reaction times at a constant concentration of *n*-butyllithium (2.5 equiv) were compared for both pristine and mechanochemically activated Mn_3_O_4_. Lithiation of Mn_3_O_4_ with the strong base *n*-butyllithium was carried out following the method described in earlier publications.^19,68^ A rotational speed for mechanochemical activation of 400 rpm was selected as the starting point since this results in a continuous reduction of the crystallite size up to a milling time of 2 h. After drying the samples, PXRD was recorded, and the phase composition was determined by Rietveld refinement.

**Figure 6:**
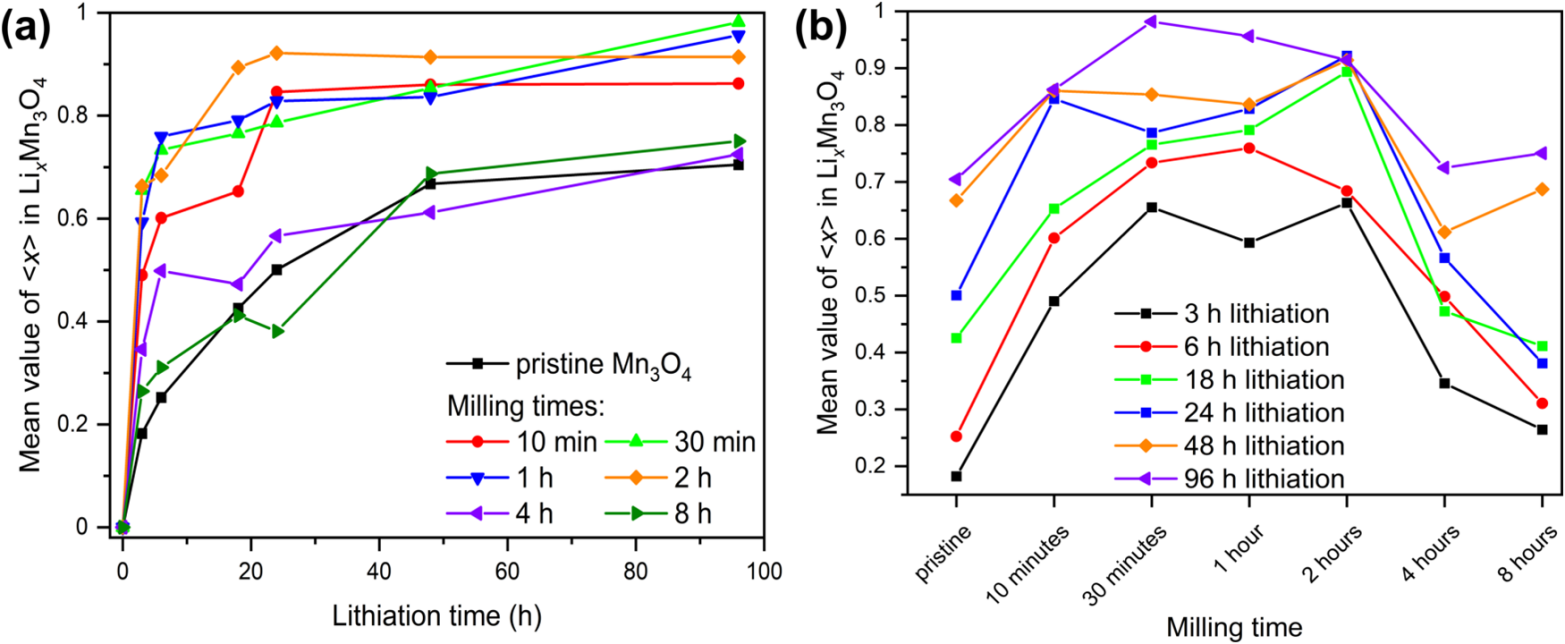
Mean value ⟨*x*⟩ of Li_*x*_Mn_3_O_4_ determined by Rietveld refinement as a function of different milling times at a rotational speed of 400 rpm and time of lithiation. Lithiation was performed with 2.5 equiv *n*-butyllithium in *n*-hexane. The two figures show the same results, in (a) the evolution of the lithium content of the individual phases can be better observed, and (b) allows a better comparison of the lithium content of the different activated samples with the same lithiation times. The lines in the figure connecting the measured points are only orientation aids for the eye.

In the first part of the study, the mean value of ⟨*x*⟩ in the obtained product (Li_*x*_Mn_3_O_4_) after different lithiation times was analyzed as a function of grinding time. Figure 6a illustrates the progression of lithiation degree of the individual samples with a certain grinding time and Figure 6b allows a better comparison of the individual lithiation times of the samples mechanochemically activated for different times. Since the transition state only occurs at a low degree of lithiation, the formulation LiMn_3_O_4_ is used below instead of Li_*x*_Mn_3_O_4_ as a simplified description of lithiated Mn_3_O_4_.

Milling Mn_3_O_4_ for 10 min at 400 rpm resulted in a steep increase of the mean value ⟨*x*⟩ of Li_*x*_Mn_3_O_4_ compared to the pristine product. For example, after 3 h lithiation ⟨*x*⟩ in the unmilled sample was just below 0.2, while in the mechanochemically activated sample for 10 min, it was approximately 0.5. For longer lithiation times, there seems to be a threshold value/maximum of around 0.95 for the mean value ⟨*x*⟩ of Li_*x*_Mn_3_O_4_ under these conditions. The decreased particle size leads to shorter diffusion path lengths, which may explain the increased mean value ⟨*x*⟩ of Li_*x*_Mn_3_O_4_ for all milling times and short lithiation times up to 18 h. However, the lower amount of Li_*x*_Mn_3_O_4_ (similar yields as for the unground sample) at long lithiation times for the two longest milled samples (4 and 8 h) could be due to the formation of agglomerates, as observed in the SEM images and associated longer diffusion paths. Moriga et al. found that prolonged milling leads to a decrease in Li^+^ ionic diffusion in lithium cobalt oxides.^69^ They attribute it to an excessively high concentration of defects and/or disorder, impeding ionic diffusion. A moderate number of defects and/or disorder enhances ionic diffusion. During ball milling of ZnO or TiNb_2_O_7,_ the specific surface area initially increases, passing through a maximum, then decreases at prolonged milling times. This decrease is ascribed to agglomerate formation, reducing the system’s excess free energy.^70,71^ To investigate whether agglomerate formation reduces lithium intercalation, wet grinding with *n*-pentane at low rotational speed (200 rpm, 30 min) was performed on activated powder (400 rpm, 4 h). After solvent evaporation, lithiation experiments were prepared for 6 h, 24 h, and 96 h. The lithiation levels were compared to heavily milled samples (400 rpm, 4 h). A slight increase in lithiation degree was observed due to wet grinding, suggesting agglomerate breakup enhances lithium intercalation. The exact values and the corresponding PXRD diagrams are provided in the SI (Figure S14 and Table S6). The optimum in mechanochemical activation time for enhancing lithium intercalation in Mn_3_O_4_ is 30 min under these tested conditions. Shorter and longer activation led to lower lithium contents (Figure 6b). However, mechanochemical activation between 10 min and 2 h resulted in relatively high lithium contents. Prolonged milling times likely led to agglomerate formation, hindering lithium intercalation. The lithium diffusion path is hindered for both short and prolonged activation times. At short milling durations, the presence of large particles leads to a low degree of lithium intercalation. Conversely, extended milling times result in agglomerate formation, impeding lithium intercalation. Consequently, an optimal milling time exists for efficient lithium intercalation into Mn_3_O_4_. Deviations from this optimal duration, either shorter or longer, result in reduced lithium intercalation. Higher lithium contents can be achieved by employing more *n*-butyllithium equivalents and/or performing the reaction under reflux conditions.

For selected samples, ICP-MS was used to determine lithium content in addition to Rietveld refinement. The results obtained fit well in a series with the Rietveld analysis, but the values determined via ICP-MS are always somewhat higher. This is probably because with ICP-MS the ratio of lithium to manganese was determined, and despite intensive washing, there is still lithium present, for example, in the form of lithium hydroxide, lithium carbonate or another lithium species adhering to the surface of the particles, which is not intercalated. With Rietveld refinement only, the intercalated lithium is determined. The determined values are shown in the SI (Table S7).

**Figure 7:**
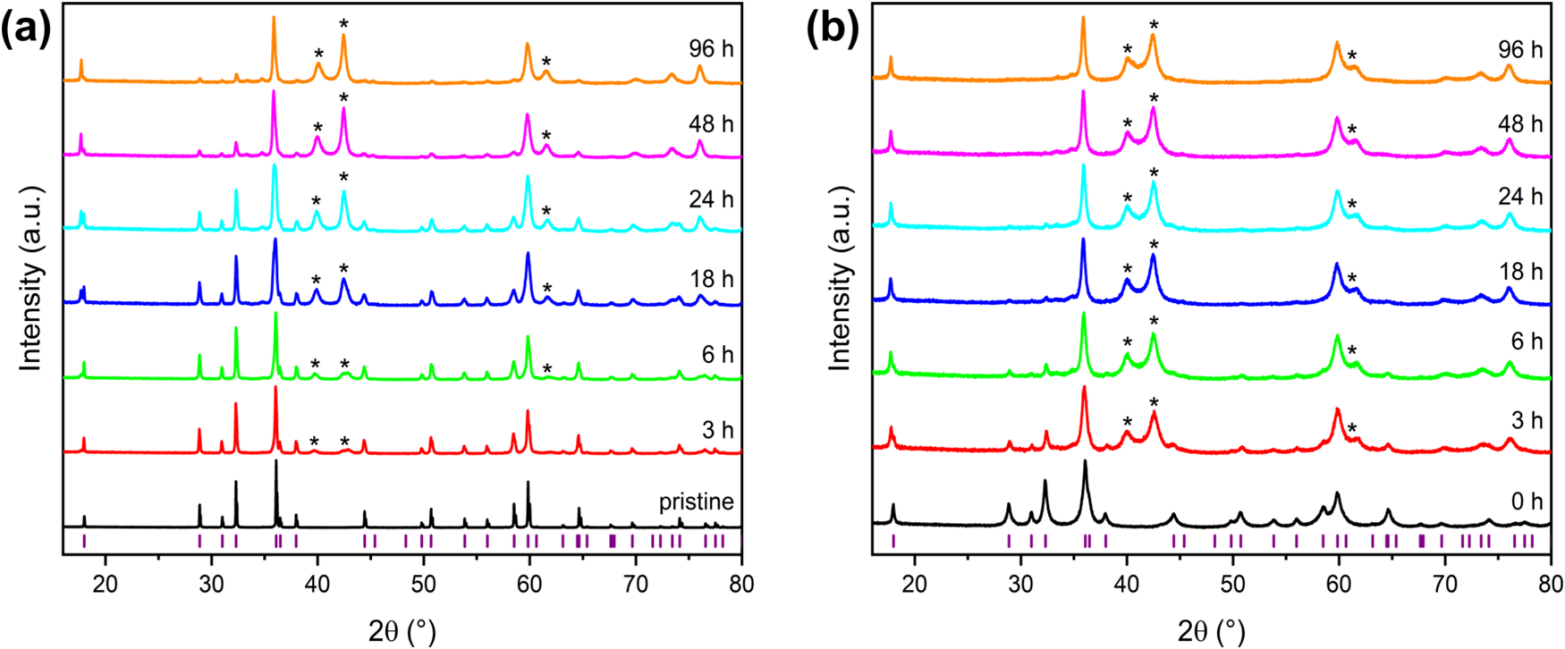
Enlarged section of the PXRD patterns of the obtained products, when pristine (a) and milled (b) Mn_3_O_4_ (30 min, 400 rpm) was lithiated with *n*-butyllithium (2.5 equiv) in *n*-hexane for different times. The purple lines indicate the *hkl*s of Mn_3_O_4_. The asterisks mark the characteristic reflections of LiMn_3_O_4_.

The PXRD patterns for the products obtained from lithiating pristine Mn_3_O_4_ with 2.5 equiv *n*-butyllithium initially resemble the starting material. However, with prolonged lithiation times, the typical reflections of LiMn_3_O_4_ at 40° 2θ and 60° 2θ become evident, indicating an increase in the LiMn_3_O_4_ phase fraction (Figure 7a). For the Mn_3_O_4_ sample (Figure 7b), the characteristic LiMn_3_O_4_ reflections are clearly visible even at shorter lithiation times after mechanochemical activation. Rietveld refinement confirms a higher phase fraction of LiMn_3_O_4_ compared to the pristine sample. This behavior is observed for all ground samples up to 2 h of milling time. Longer grinding times result in a lower proportion of the lithiated product for the same lithiation times compared to shorter milling durations. No side phases or byproducts were detected after milling (Figures S15–S19).

**Figure 8:**
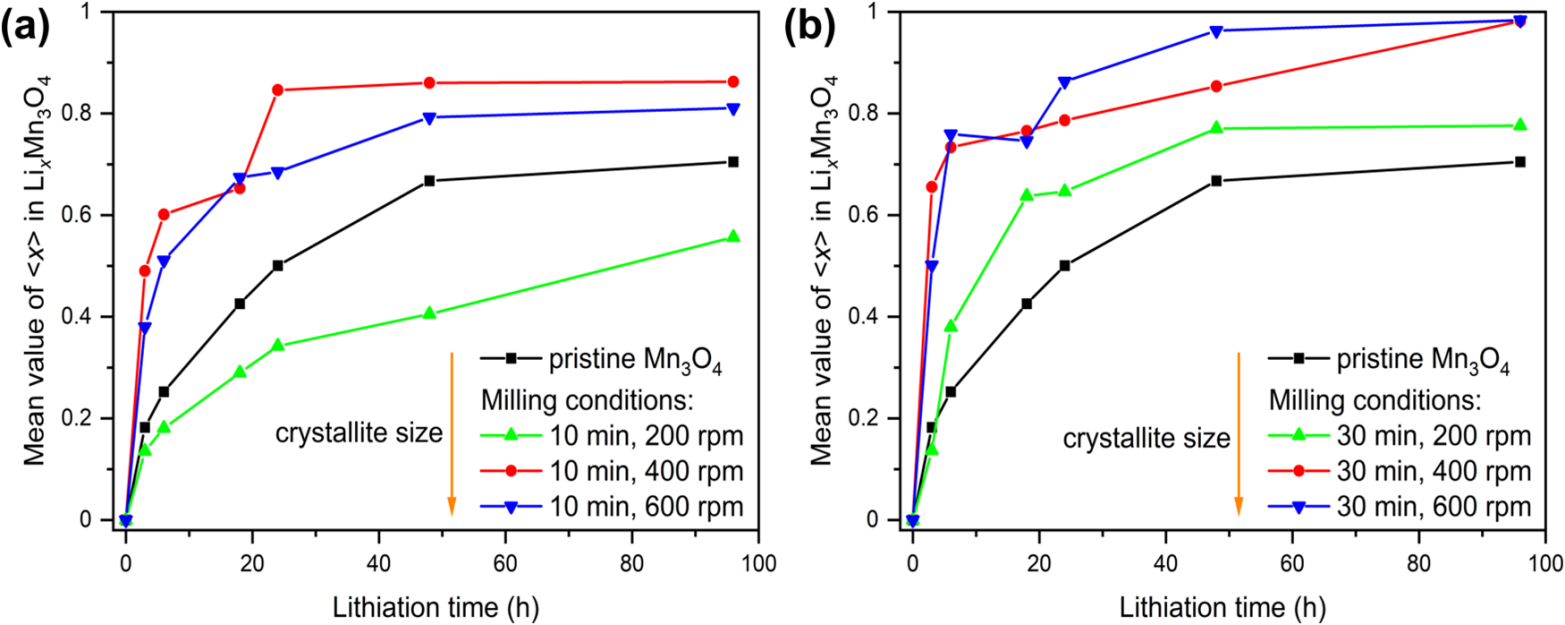
Mean value ⟨*x*⟩ of Li_*x*_Mn_3_O_4_ depending on different rotational speeds at two different times (10 min (a) respectively 30 min (b)) and duration of lithiation. The orange arrow shows the evolution of the crystallite size under the grinding conditions used. Lithiation was performed with 2.5 equiv *n*-butyllithium in *n*-hexane. The amount of Li_*x*_Mn_3_O_4_ was determined by Rietveld refinement. The lines in the figure connecting the measured points are only orientation aids for the eye.

The effect of the rotational speed on mechanochemical activation and lithiation kinetics was investigated by varying the rotational speed at a constant milling time. The Mn_3_O_4_ was milled for 10 min at 0 (pristine), 200, 400 and 600 rpm, followed by lithiation with 2.5 equiv *n*-butyllithium in absolute *n*-hexane for different times. The 10 min milling time was chosen because a noticeable difference in the amount of lithiated product was observed at 400 rpm. The determined crystallite sizes of the milled Mn_3_O_4_ shown in Figure 1b demonstrate the expected larger reduction in crystallite size with higher rotational speed at constant milling times. The crystallite size (>500 nm) of the starting material was reduced to 347(23) nm, 90(9) nm, and 34(3) nm at 200, 400, and 600 rpm, respectively. No major differences are observed between the two activations at 400 and 600 rpm for 10 min and, in both cases the amount of LiMn_3_O_4_ was higher compared to the pristine and the product activated at 200 rpm (Figure 8a). This suggests that at short grinding times and high rotational speeds, lithiation is facilitated equally well in both cases. The lowest yield of LiMn_3_O_4_ is obtained for activation at 200 rpm for 10 min, even lower than the yield of the unground sample. At the low rotational speed, an effect seems to impede lithiation, possibly due to particle size. At 200 rpm, the particles are still very large, and grinding leads to strain, hindering the intercalation of lithium into the particle core, resulting in lithiation primarily of the outer shell. Cabana prepared LiMn_2_O_4_ via high pressure synthesis and Na/Li ion exchange and demonstrated that surface defects blocked the cation transfer in LiMn_2_O_4_ prepared via high pressure synthesis.^72^ In the case of unground Mn_3_O_4_, a more crystalline structure without a significant amount of strain is present, allowing better lithium intercalation. Mechanochemical activations at rotational speeds of 400 rpm or more result in a sharp diminution of the crystallite size and increased formation of defect sites, facilitating the diffusion of lithium into the particle core.

We investigated the rotational speed’s influence at a fixed 30 min milling time (Figure 8b). No significant difference in the determined fraction of LiMn_3_O_4_ was observed between the 400 and 600 rpm activations. At both speeds, a significantly higher lithiated product was observed compared to the unground reactant. Activation at 200 rpm with the different lithiation durations results in slightly higher LiMn_3_O_4_ amount than the unground reactant, but lower than the two higher rotational speeds. The corresponding PXRD diagrams are in the SI (Figures S20–S23).

At a specific rotational speed and grinding duration, the crystallite size decreases while the defect concentration increases simultaneously. This facilitates lithium diffusion into the particle. After 30 min of grinding at 200 rpm, the LiMn_3_O_4_ phase fraction is larger compared to 10 min, indicating prolonged low-speed grinding enhances lithium intercalation, likely by reducing crystallite size and elevating defect concentration.

All organolithium compounds exhibit strong basicity. For the compounds employed herein, their relative basicity follows the order dictated by the p*K*_a_ (in parentheses) of their conjugate acids:
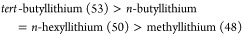


The solvation shell surrounding lithium species could influence lithiation. The number of coordinated solvent molecules varies with the organolithium reagent and solvent employed (Table S8).^73^

To investigate the influence of solvent and lithiation reagent, various solvents (*n*-hexane, THF, diethyl ether) and lithiation agents (*tert*-butyllithium, *n*-butyllithium, methyllithium and *n*-hexyllithium) were employed. The obtained products were analyzed. When using methyllithium, the solvent was changed to THF due to its insolubility in *n*-hexane.^73^ The lithium content of the products obtained from milled Mn_3_O_4_ is presented in Table 2. Corresponding values for pristine Mn_3_O_4_ under identical reaction conditions are provided in the SI (Table S9).

**Table 2:** Mean Value of ⟨*x*⟩ in Li_*x*_Mn_3_O_4_ when Using Different Lithiation Agents for Lithiation (*tert*-Butyllithium, *n*-Butyllithium, or Methyllithium) of Milled Mn_3_O_4_ (30 min, 400 rpm) as a Function of the Different Solvents Used

	*n*-hexane		THF		diethyl ether	
time (h)	*tert*-BuLi	*n*-BuLi	*n*-BuLi	MeLi	*n*-BuLi	MeLi
3	0.438	0.655	0.305	0.179	0.727	0.192
6	0.607	0.734	0.339	0.268	0.761	0.297
18	0.587	0.766	0.285	0.409	0.894	0.495
24	0.679	0.787	0.322	0.480	0.909	0.513
48	0.671	0.854	0.339	0.601	0.939	0.681
96	0.675	0.982	0.324	0.707	0.925	0.761

Irrespective of the lithiation reagent, milled Mn_3_O_4_ exhibited a higher lithiation degree under the tested conditions. Employing *tert*-butyllithium as the lithiation agent for Mn_3_O_4_ yielded lower lithiation degrees compared to *n*-butyllithium when using the same reaction parameters, as confirmed by PXRD and Rietveld refinement (Figures S24–S26). The use of *n*-butyllithium resulted in a higher yield of LiMn_3_O_4_ compared to methyllithium. However, the solvent employed during chemical lithiation can also influence the lithiation efficiency. To evaluate the solvent effect on lithiation with methyllithium, the reaction was repeated in diethyl ether (Figures S27–S31). Under identical reaction conditions, a higher proportion of lithiated Mn_3_O_4_ was obtained using diethyl ether compared to THF as the solvent. To further investigate, *n*-butyllithium was employed as the lithiating reagent in both THF and diethyl ether. The results showed that only a small fraction of Mn_3_O_4_ was lithiated in THF, whereas a significantly higher proportion was lithiated in diethyl ether, approaching the level achieved with *n*-butyllithium in *n*-hexane (Figures S32–S36). *n*-Hexyllithium was investigated as an alternative lithiation reagent. Compared to *n*-butyllithium, it exhibited a slightly lower yield of LiMn_3_O_4_, except for the longest lithiation time. While *n*-butyllithium showed no further increase in the lithiation degree after 24 h, *n*-hexyllithium demonstrated a steady increase over the entire lithiation period. However, milling was only carried out for 10 min at 400 rpm (Figures S37–S39). The highest lithiation degrees are achieved using *n*-butyllithium with *n*-hexane as solvent; *n*-hexyllithium yields comparable results. Neither basicity nor the number of coordinated solvent molecules appear crucial for lithiation, as *n*-butyllithium consistently outperforms other bases. However, when using THF as solvent, deprotonation of the solvent by *n*-butyllithium may occur as a side reaction under the reaction conditions, potentially hindering the desired lithiation.^74^

Lithiation was also attempted using lithium sulfide (Li_2_S) with a 5-fold excess, stirred for 96 h at room temperature under argon in acetonitrile. Rietveld refinement revealed no lithiation of mechanochemically activated Mn_3_O_4_ (10 min, 400 rpm) under these conditions (Figure S40).

Electrochemical impedance measurements can provide information about the density of defects in a material, especially resistance and conductivity. An impedance spectrum may be described by an equivalent circuit. Common elements in such a circuit are resistors *R*, capacities *C* and constant phase elements CPE. The dependency of the impedance *Z* of a CPE on the frequency may be written as (eq 1)^75^
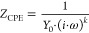
1The exponent *k* may be any value out of the interval [0,1]. There are two limit cases: for *k* = 0 the CPE is the same as a resistor and for *k* = 1 the CPE is the same as a capacitor.

The conductivity σ depends on the frequency ω. The real part of the conductivity may be written as (eq 2)
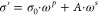
2in which σ_0_ is the conductivity of direct current and *p* and *s* are positive exponents.^76^ The low-frequency dispersion (*lfd*) is mainly influenced by the parameter *p*, while the high frequency dispersion (*hfd*) reveals the parameter *s*. The *lfd* is caused by hopping of carriers over a longer path until the path is blocked by any reason. The *hfd* additionally allows hopping along short paths involving an increasing conductivity. The value of *p* is close to zero and often is assumed to be equal to 0.^77,78^ The value of *s* is often within the range of 0.5–0.9.^79^ A is a material depending constant. The frequencies between those, which may not clearly be assigned to the *lfd* or *hfd* will be called “intermediate region”.

Only few data about the conductivity σ of Mn_3_O_4_ may be found in literature, which are often old and do not provide direct values or discuss the behavior at high temperatures.^80−83^ Dhaouadi et al. provide σ_0_ = 1.87 × 10^–5^ S/m for nanocrystalline, hydrothermally synthesized Mn_3_O_4_, Verwey and de Boer give σ_0_ ≈ 3.5 × 10^–5^ S/m.^77,83^

Normally, pellets are pressed from the material to be tested and then sintered and measured. However, sintering leads to the healing of the defects and the increase of the crystallite size in our samples and must therefore be omitted, which makes the pellets very fragile and brittle, which has led to the fact that only a few samples could be measured. The compaction of some of the pellets was calculated from the ratio of the compacted bulk density and the theoretical bulk density. The compaction was between 65 and 70% and may be assumed to form a systematic, nearly constant influence on the impedance measurements. The Nyquist-plots of all the samples of Mn_3_O_4_ showed fine semicircles (Figure S41a), which could be fitted by a parallel circuit of a CPE element and a resistor *R*_1_, which was in a serial circuit with an additional resistor *R*_0_. The exponent *k* (eq 1) was found to be >0.96, which is close to an ideal capacitor (*k* = 1). Lower values of *k* might be caused by double layer capacitance, caused by ions moving close to the contacting electrodes.^84^ This is not the dominant effect for Mn_3_O_4_. The behavior is mainly determined by hopping of electrons between Mn^2+^ and Mn^3+^ cations. Using a capacitor *C*_1_ instead of a CPE element also provided a reliable fit, but a serial circuit of a resistor and two or three RC-circuits was used for the final fit (Figure S41b, *n*_RC_ = 2 or 3). A total resistance *R*_RC_ and a total capacity *C*_RC_ (Table 3) was calculated from all of the RC-circuits.

**Table 3:** Number *n*_RC_ of RC-circuits, Resistance *R*_RC_, Capacity *C*_RC_, Conductivity σ_0_, Low Frequency Dispersion Exponent *p*, Lithium Content *x*, Milling Time *t*_bm_, Rotational Speed for Ball Milling *rs* and, Time for Lithiation *t*_Li_ of Some Samples of Mn_3_O_4_ and Li_*x*_Mn_3_O_4_

molecular formula	*x*	*t*_bm_ (h)	*rs* (rpm)	*t*_Li_ (h)	*n*_RC_	*R*_RC_ (MΩ)	*C*_RC_ (pF)	σ_0_ (S/m)	*p*
Mn_3_O_4_									
	0	0.5	400	0	2	30.9	181	1.48 × 10^–6^	0.002
	0	0.5	600	0	2	17.1	182	2.33 × 10^–6^	0.003
	0	4	400	0	2	9.9	183	5.02 × 10^–6^	0.001
Li_*x*_Mn_3_O_4_									
	0.71	0	0	96	3	7.28	224	4.24 × 10^–6^	n.a.
	0.73	0.5	600	6	3	0.54	194	9.35 × 10^–5^	n.a.
	0.61	4	400	48	3	1.12	205	4.05 × 10^–5^	n.a.

The more intensive the mechanical treatment of Mn_3_O_4_ has been the lower the resistance *R*_RC_ of the Mn_3_O_4_ sample is. The resistance and also the resistivity of the sample milled for 30 min at 400 rpm (Figure S41a, green line) is nearly three times as big as the resistivity of the sample milled for 4 h at 400 rpm (Figure S41a, black line).

The Nyquist-plots of the Li_*x*_Mn_3_O_4_ samples all show a less perfect semicircle (Figure S41b) and a lower *R*_RC_ than the corresponding unlithiated samples. For these samples the hopping of Li^+^ is probably responsible for the lower resistivity. But, of course, the electron hopping between Mn^2+^ and Mn^3+^ still happens. The result is a severe overlap of different semicircles of both effects. But also, formation of double layer capacities by the migration of Li^+^ and inhomogeneous lithiation states may be responsible for additional “broadening” of the semicircles. To get an acceptable fit three or four RC-circuits are applied (Table 3). The resistance of the sample milled for 30 min is much lower than for the sample without activation step by a factor greater than ten. At a first glance it seems obscure, that the resistance of the heavily milled sample is higher than of the moderately milled sample, opposite to the behavior of the corresponding samples of Mn_3_O_4_. However, the heavily milled sample has a lower lithium content, although the time for lithiation was eight times longer. The mobility of the Li^+^ ions appears to be responsible not only for the process of lithiation, but also for the hopping of the ions under an electric field.

**Figure 9:**
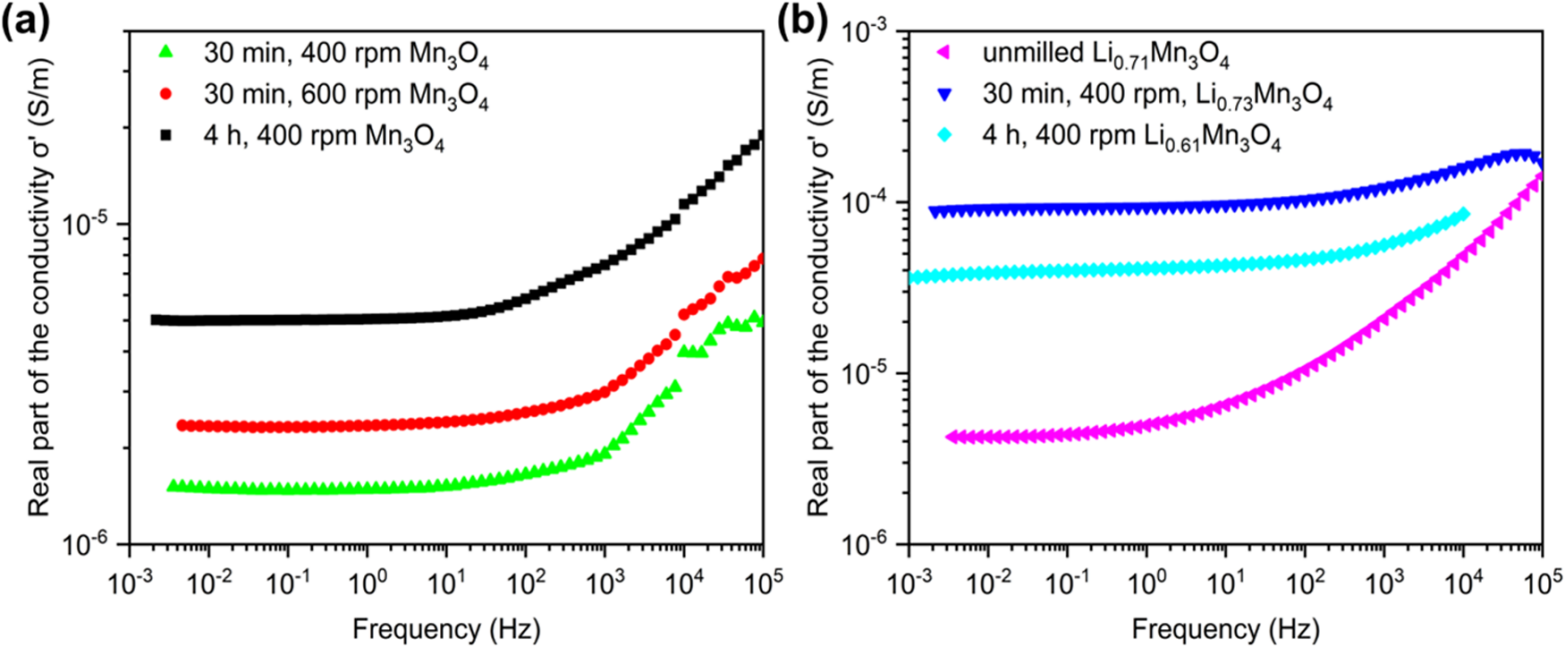
Frequency dependent real part of the conductivity at room temperature of Mn_3_O_4_ samples mechanochemically activated under different conditions (a) and of unmilled and milled Li_*x*_Mn_3_O_4_ samples (b).

The *lfd* shows a nearly constant value for the conductivity up to a frequency of about 10 Hz, but a small incline is visible (Figure 9). The corresponding exponent *p* in eq 2 was estimated to be within the range of 0.001–0.003. Due to this small incline the values of σ_0_ are only less than 0.5% lower than the corresponding values calculated from *R*_RC_ derived by fitting the semicircles of the impedance.

For the *hfd*, the graph provides a drastic increase of σ′, but a similar slope at frequencies higher than about 1 kHz exists. Graphically a corresponding exponent *s* was estimated to lay in the range of 0.45–0.55. These values are similar to exponents reported in literature for other materials.^79^

The sample milled at 400 rpm for 4 hours showed more deviation from the simulated graph than other Mn_3_O_4_ samples. The line shapes of PXRD reflections for this sample revealed smaller crystallites, more strain, and a more severe asymmetry than both others. Strain and peak asymmetry are hints to inhomogeneity of the material. Perhaps such inhomogeneity is the reason for a less proper fit to the simulated σ′(ω).

The conductivity increases with increasing mechanical load during milling. The highest value of σ_0_ found is about 5 × 10^–6^ S/m. This is more than three times as high as the lowest value listed in Table 3 for Mn_3_O_4_. An uncertain impedance spectrum, based on another sample not considered here, give rise to a conductivity of Mn_3_O_4_ below 1 × 10^–7^ S/m before activation by hebm. The values provided by literature are much higher but are very old or are based on a completely different synthesis route.^77,83^ Carbon contamination would increase the conductivity of a sample, which may be the case for some of the values given in literature.

As already discussed for the values of *R*_RC_ of the lithiated samples there is a corresponding maximum of the conductivity within the *lfd*, represented by the values of σ_0_. The maximum observed is about 1 × 10^–4^ S/m. The regions of the *lfd* and *hfd* with nearly constant incline, represented by the parameters *p* and *s*, seem to be smaller than for Mn_3_O_4_. The bigger intermediate range of frequencies probably is caused by the inhomogeneity mentioned above. The values of *p* tend to be higher than for Mn_3_O_4_ and the values of *s* tend to be a little bit lower. Within the *hfd* σ′ seem to have a trend to converge to a similar value for very high frequencies for all three samples. To confirm this hypothesis more complex research is necessary, implementing more samples and material synthesized via different routes and scanned at different temperatures.

## Conclusions

In this study, the effects of high-energy ball milling on coarse-grained Mn_3_O_4_ were investigated, highlighting the evolution of crystallite size and the resulting consequences for lithiation with different lithiation reagents. We used coarse-grained Mn_3_O_4_ obtained by high-temperature synthesis as starting material and ground it in a ball mill at different durations and speeds. Through a comprehensive analysis including Rietveld refinement of the PXRD data, Raman spectroscopy, TEM and SEM images, we were able to demonstrate a reduction in crystallite size as a function of the extent of milling. Furthermore, we investigated the influence of mechanochemical activation of Mn_3_O_4_ on its lithiation behavior and the formation of LiMn_3_O_4_ by kinetic studies. Remarkably, mechanochemical activation led to an increased yield of LiMn_3_O_4_, which was particularly evident for shorter milling times. However, longer grinding times only led to a marginal increase in yield, probably due to agglomeration effects. Furthermore, the choice of lithiation reagents and solvent in the chemical lithiation reaction had a significant influence on the obtained yield of LiMn_3_O_4_, lithiation of mechanochemically activated Mn_3_O_4_ for 30 min at a rotational speed of 400 rpm with *n*-butyllithium in *n*-hexane gives the best results under these conditions. In terms of conductivity, coarse-grained Mn_3_O_4_ exhibited a conductivity of less than 1 × 10^–7^ S/m, while mechanochemically activated Mn_3_O_4_ achieved a significantly higher conductivity of up to 5 × 10^–6^ S/m. After lithiation, the conductivity of the samples increased considerably and reached values of up to 1 × 10^−4^ S/m. Interestingly, very long grinding times did not lead to a significantly lower increase in conductivity compared to shorter times, reflecting the performance observed during lithiation. These results highlight the intricate relationship between mechanochemical activation, lithiation behavior and conductivity in Mn_3_O_4_-based systems and provide valuable insights for the development of advanced energy storage materials.

## Supporting Information Available

Characterization of the obtained materials; including structure refinement parameters, site occupation factors; crystallite size, PXRD, TEM images, SEM images; physisorption isotherms; pressure and temperature curves during MCA; lithiation degree of the individual phases and relative amounts of the individual phases; ICP-MS; mean value ⟨*x*⟩ of Li_*x*_Mn_3_O_4_ depending on different lithiation reagents and solvents and Nyquist plots (PDF)
