# Supplementary material for: Mechanochemical Activation of Mn3O4: Implications for Lithium Intercalation
Source: Inorg Chem. 2025 Mar 14;64(13):6420–33. doi: 10.1021/acs.inorgchem.4c04660 (PMC11979893; doi:10.1021/acs.inorgchem.4c04660)
Supplement: Supplementary file 1 — ic4c04660_si_001.pdf [file ic4c04660_si_001.pdf]

# Supporting Information

# Mechanochemical Activation of $Mn_3O_4$: Implications for Lithium Intercalation

*Tobias Benjamin Straub*[a], *Robert Haberkorn*[a] *and Guido Kickelbick*[a]*

[a] Saarland University, Inorganic Solid-State Chemistry, Campus, Building C4.1, 66123 Saarbrücken, Germany. E-mail: guido.kickelbick@uni-saarland.de

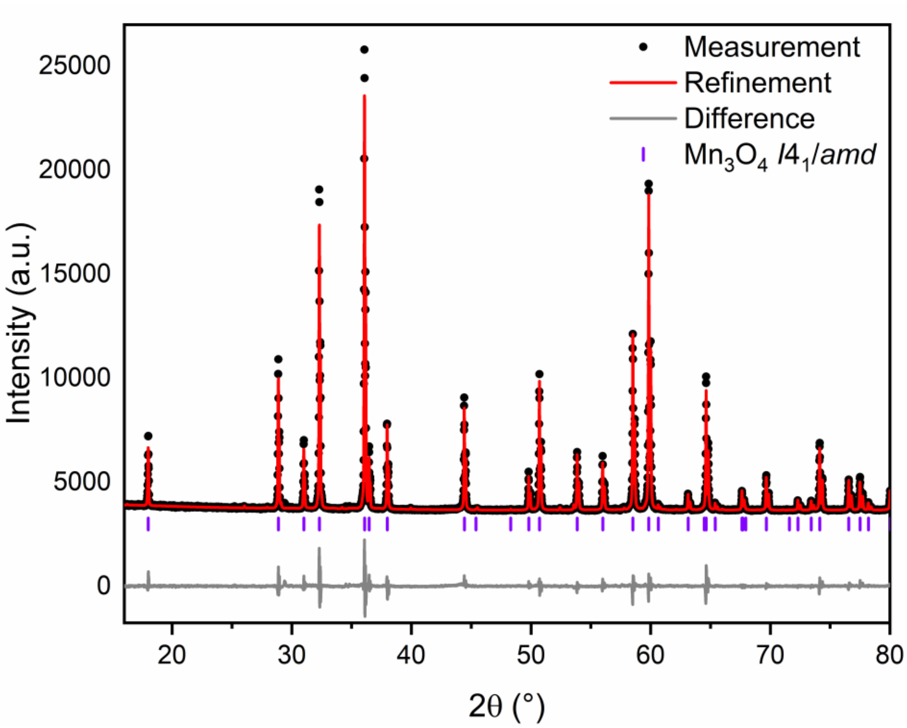


**Figure S1.** Enlarged section of the Rietveld plot of $Mn_3O_4$ obtained via solid state reaction. $Mn_3O_4$ in space group $I4_1/amd$ with $a$ = 576.29(1) pm, $c$ = 946.80(1) pm and $V$ = 0.314444(4) $nm^3$. Refinement parameters: number of independent parameters = 28, $R_{wp}$ = 8.68%, $R_{exp}$ = 4.93%, GOF = 1.76. The purple lines indicate the *hkl*s of $Mn_3O_4$. From the Rietveld refinement a crystallite size larger than 500 nm was determined.

**Table S1.** Structure Refinement Parameters of $Mn_3O_4$ and $LiMn_3O_4$.

| $Mn_3O_4$ (Figure S1) and $LiMn_3O_4$ (Figure 4) | | |
|---|---|---|
| Source | Bruker D8 ADVANCE (Laboratory X-ray) | |
| Chemical formula | $Mn_3O_4$ | $LiMn_3O_4$ |
| Formula weight | 228.8 | 235.8 |
| Temperature | RT | |
| Pressure | ambient | |
| Wavelength for constant wavelength | 154.0596 and 154.4308 pm | |
| Crystal system | tetragonal | tetragonal |
| Space group | $I4_1/amd$ (141) | $I4_1/amd$ (141) |
| *a* | 576.29(1) pm | 605.39(2) pm |
| *b* | *a* | *a* |
| *c* | 946.80(1) pm | 898.12(5) pm |
| *V* | 0.31444 $nm^3$ | 0.32915 $nm^3$ |
| *Z* | 4 | 4 |
| *d*-space range | 0.89 - 12.62 Å (7 - 120° 2$\theta$) | |
| $\chi^2$ | 3.11 | 2.44 |
| $R_p$ | 6.72 | 6.36 |
| $R_{wp}$ | 8.69 | 8.06 |
| $R_{exp}$ | 4.93 | 5.16 |
| Definition of *R*-Factors | $R_p = \frac{\sum\lvert Y_0 - Y_c\rvert}{\sum Y_0}$; $R_{wp} = \left(\frac{\sum w(Y_0 - Y_c)^2}{\sum w{Y_0}^2}\right)^{\frac{1}{2}}$; $R_{exp} = \left(\frac{M - P}{\sum w{Y_0}^2}\right)^{\frac{1}{2}}$ | |
| GOF (Goodness of fit) | 1.76 | 1.56 |

**Table S2.** Site occupation factors (*sof*) of the reference samples for $Mn_3O_4$ and $LiMn_3O_4$.

| Wyckoff Position | Atom | $Mn_3O_4$ Occupation | $LiMn_3O_4$ Occupation |
|---|---|---|---|
| 4*a* | $Li^{1+}$ | 0 | 0 |
| | $Mn^{2+}$ | 1 | 0 |
| 8*c* | $Li^{1+}$ | 0 | 0.5 |
| | $Mn^{2+}$ | 0 | 0.5 |
| 8*d* | $Mn^{2+}$ | 0 | 0.5 |
| | $Mn^{3+}$ | 1 | 0.5 |
| 16*h* | $O^{2-}$ | 1 | 1 |

The coordinates of the Wyckoff position 16$h$ were refined to (0, 0.4711(3), 0.25912(16)) for $Mn_3O_4$ and to (0, 0.4966(6), 0.2490(4)) for $LiMn_3O_4$.

Definition of the tetragonal distortion *q*

$Mn_3O_4$ is known to transform to a cubic state at high temperatures of about 1180 °C.[1] Because there is no hysteresis during heating and cooling this is a topotactical phase transition. The high temperature phase may be regarded having the structure type of the well-known cubic spinel (space group $Fd\bar{3}m$),[2] which is an $\sqrt{2} \times \sqrt{2} \times 1$ superstructure of the tetragonal spinel type. A measure *q* of the tetragonal distortion of the tetragonal lattice in comparison with the cubic state may be defined as (Equation 1)

$$q = \frac{c}{\sqrt{2} \cdot a} - 1. \qquad (1)$$

The same definition of $q$ may be used for $LiMn_3O_4$, which is a tetragonally distorted $\sqrt{2} \times \sqrt{2} \times 2$ superstructure of the rock salt structure type.

**Table S3.** Crystallite size (nm) determined by Rietveld refinement depending on milling time (min) and rotational speed (rpm).

| Milling time (min) | Crystallite size at rpm | | |
|---|---|---|---|
| | 200 | 400 | 600 |
| 0 | >500 | >500 | >500 |
| 10 | 347(23) | 90(9) | 34(3) |
| 30 | 232(9) | 60(2) | 12(1) |
| 60 | 83(16) | 38(9) | 9(3) |
| 120 | - | 10.2(0.6) | - |
| 240 | - | 8.5(5) | - |
| 480 | - | 8.9(7) | - |

Multi-fraction model in Rietveld refinement for the description of the milled $Mn_3O_4$

The crystallite size was the primary parameter used to describe the change in $Mn_3O_4$ due to milling, and the lattice parameters were considered as a secondary parameter. Strain was also taken into account but is of minor significance within a multi-fraction model and will not be discussed in this work. For this purpose, the values for *a* and *c* were constrained. For the grinding at 400 rpm and 10 minutes a value of *a* = 576.36(1) pm and a value of for *c* = 946.75(1) pm was determined if the grinding was extended to 1 hour the value of *a* and *c* was 576.47(4) pm respectively 945.56(7) pm. By using a multi-fraction model for Rietveld refinement of milled $Mn_3O_4$, better fits can be achieved as opposed to using only one fraction. For the description of $Mn_3O_4$ which was milled for 30 minutes at 400 rpm, a three-fraction model is applied. For the lattice parameters *a* and *c*, slightly different values were assumed as initial values. The plot of the corresponding Rietveld refinement is shown in Figure S2.

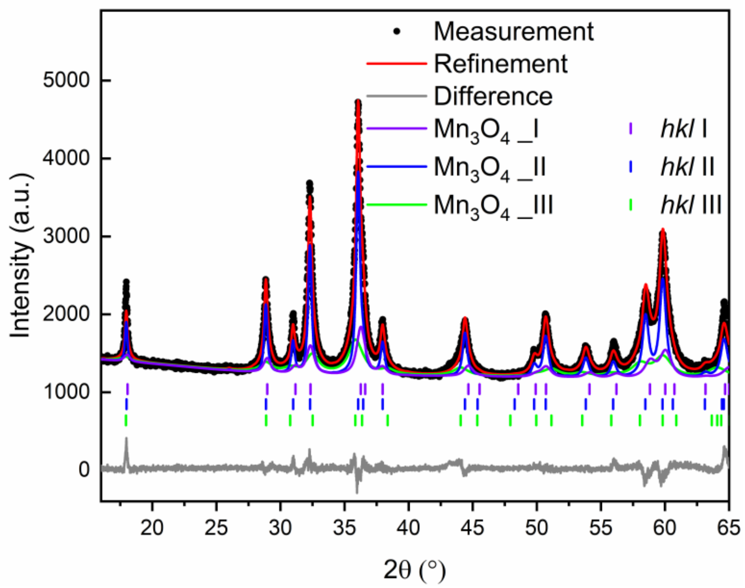


**Figure S2.** Enlarged section of the Rietveld plot of $Mn_3O_4$ milled for 30 minutes at 400 rpm. Three fractions with slightly different lattice parameters for each fraction were used to achieve a better fit.

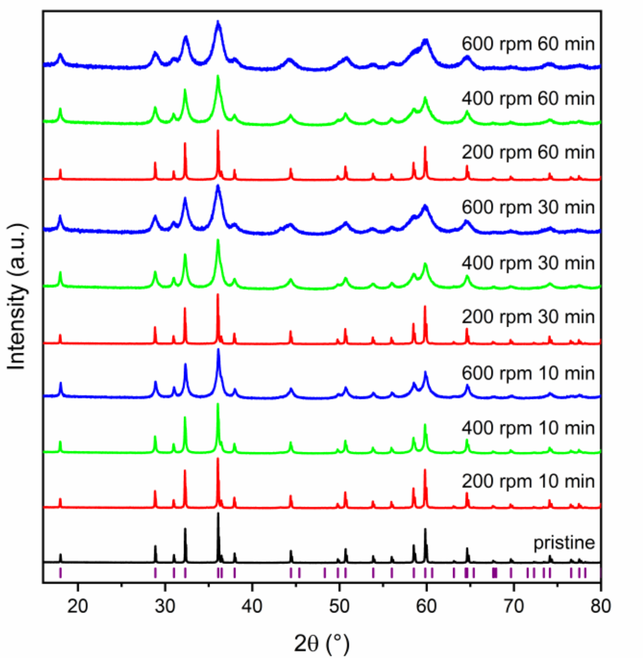


**Figure S3.** Enlarged section of the PXRD patterns of $Mn_3O_4$ milled in a 45 mL $ZrO_2$ grinding jar and 180 $ZrO_2$ milling balls (diameter of 5 mm) at different rotational speeds and different time lengths. The ball to powder ratio hereby was 23:1. The purple lines indicate the *hkl*s of $Mn_3O_4$.

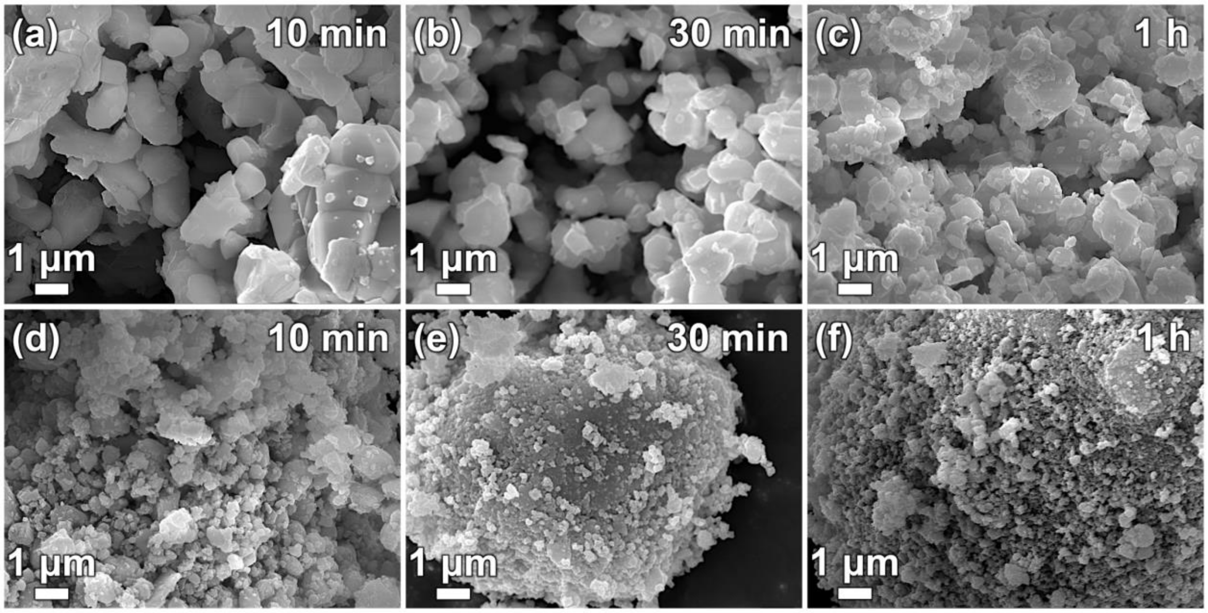


**Figure S4.** SEM images of milled $Mn_3O_4$ at different milling times, 10 minutes (a, d), 30 minutes (b, e), 1 hour (c, f) and different rotational speeds (200 rpm (a - c) and 600 rpm (d - f)). Factor of magnification 10000.

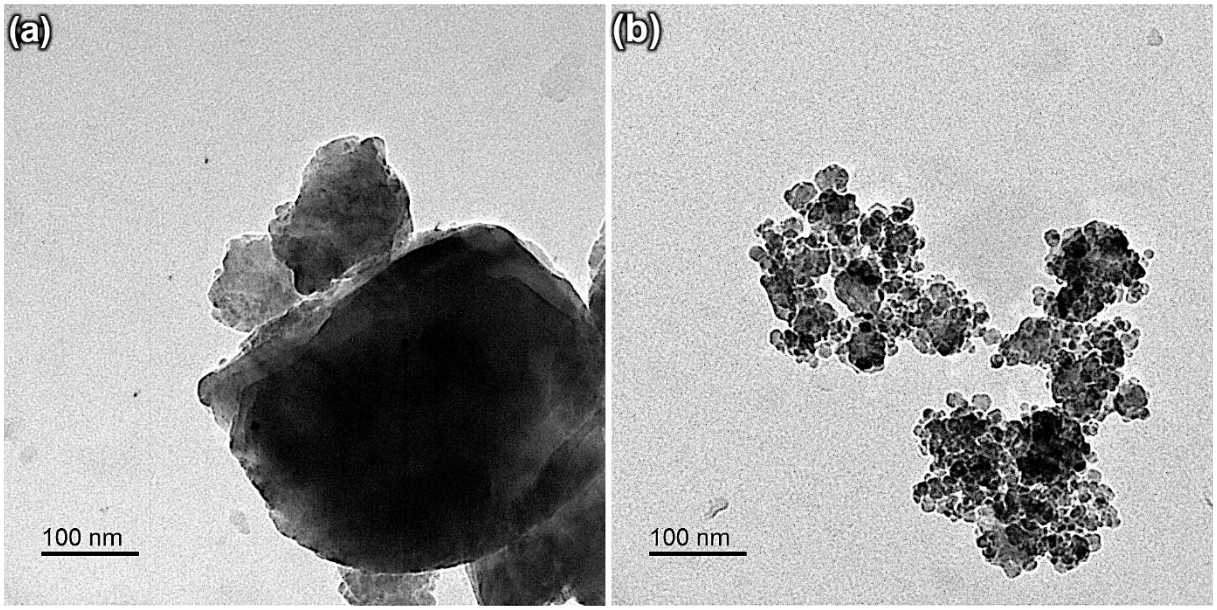


**Figure S5.** TEM images of milled $Mn_3O_4$ at 400 rpm for 10 min (a) and 8 h (b).

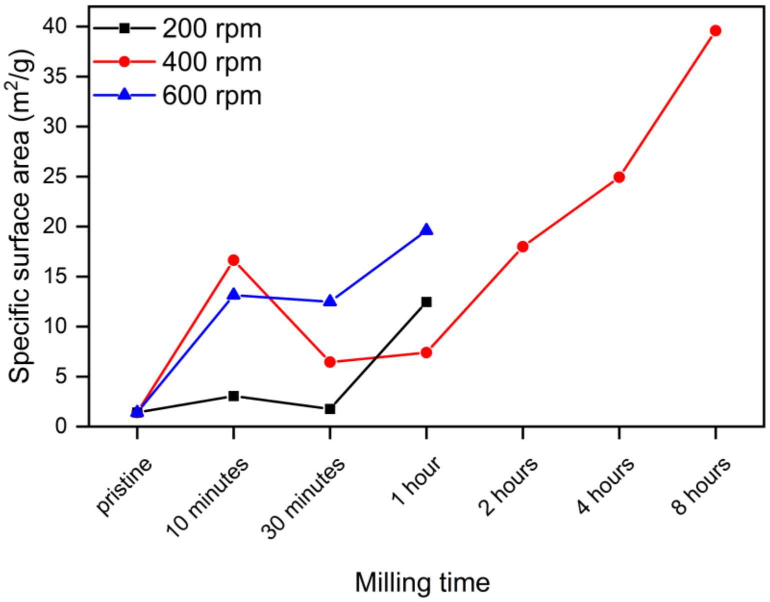


**Figure S6.** Development of the specific surface area as a function of the milling time and the rotational speed. The lines in the figure connecting the measured points are only orientation aids for the eye.

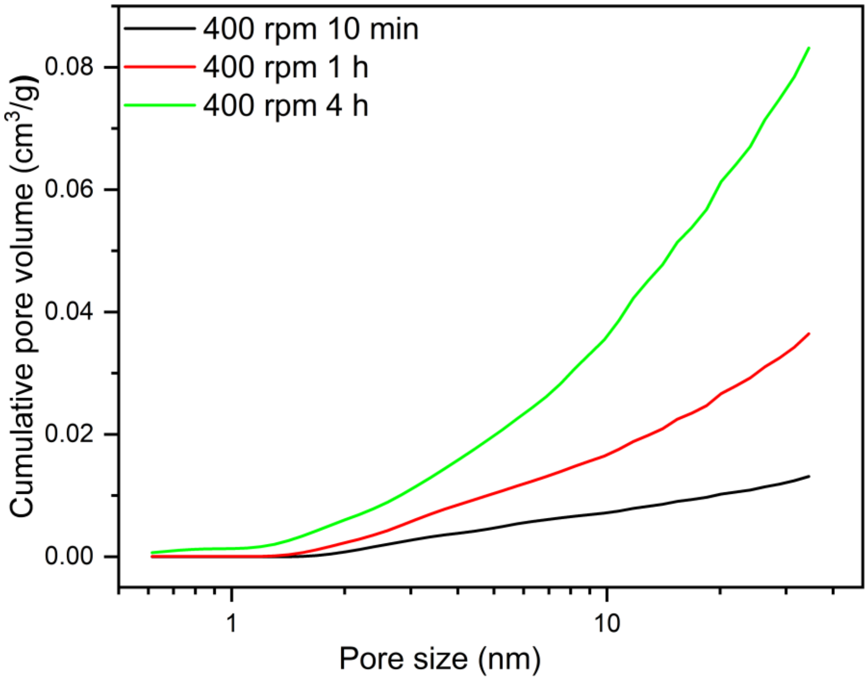


**Figure S7.** Cumulative pore volume obtained from physisorption isotherms for milled $Mn_3O_4$ at different milling times with constant rotational speed of 400 rpm.

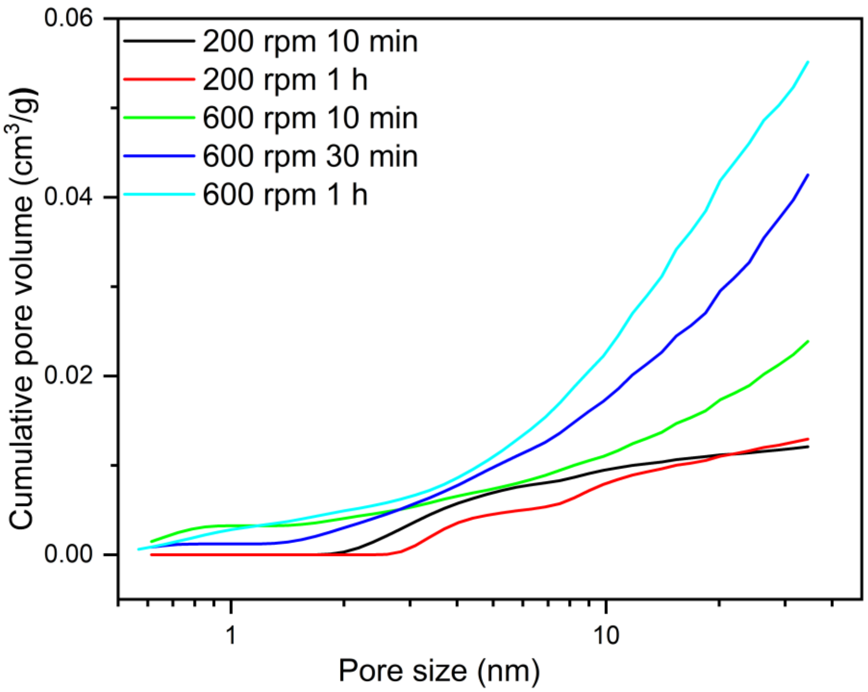


**Figure S8.** Cumulative pore volume obtained from the physisorption isotherms for milled $Mn_3O_4$ at different milling times and different rotational speeds.

**Table S4.** Specific surface area ($m^2/g$) determined by nitrogen physisorption depending on milling time (min) and rotational speed (rpm).

| Milling time (min) | Specific surface area at rpm | | |
|---|---|---|---|
| | 200 | 400 | 600 |
| 0 | 1.4 | 1.4 | 1.4 |
| 10 | 3.1 | 16.6 | 13.1 |
| 30 | 1.8 | 6.4 | 12.5 |
| 60 | 12.5 | 7.4 | 19.6 |
| 120 | - | 18.0 | - |
| 240 | - | 24.9 | - |
| 480 | - | 39.6 | - |

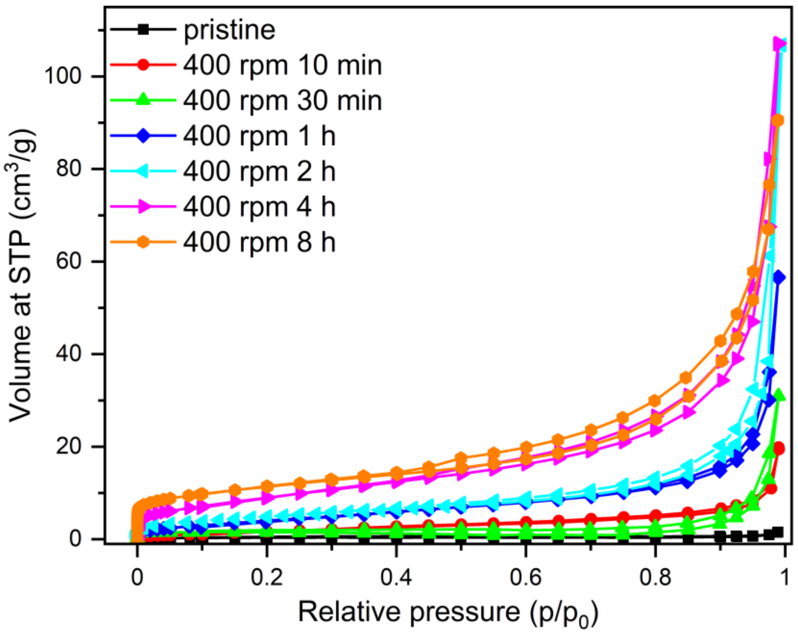


**Figure S9.** Physisorption isotherms of pristine and milled $Mn_3O_4$ at different milling times with constant rotational speed of 400 rpm.

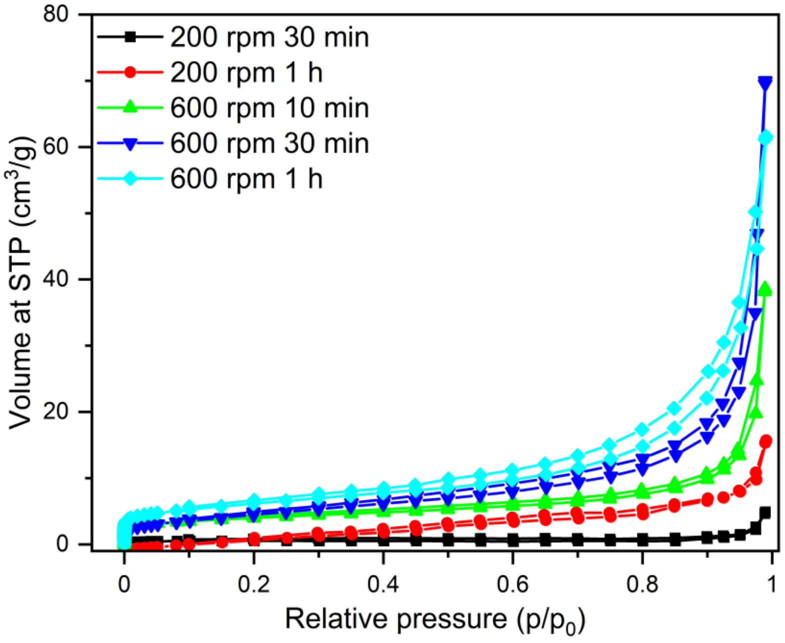


**Figure S10.** Physisorption isotherms for milled $Mn_3O_4$ at different milling times and different rotational speeds.

**Pressure and temperature profile during grinding of $Mn_3O_4$**

The experiment was conducted under an argon atmosphere. Initially, the temperature in the grinding bowl rose rapidly from 24 °C to approximately 38 °C, then remained constant throughout the grinding process (Figure S11). The pressure curve followed a similar trend, decreasing slightly from around 120 mbar to 100 mbar with prolonged grinding time. The overall pressure increase can be attributed to the temperature rise, as predicted by the ideal gas law. This loss of pressure probably is caused by a very small leakage rate of the milling jar. Consequently, the grinding of $Mn_3O_4$ did not result in significant oxygen vacancy formation.

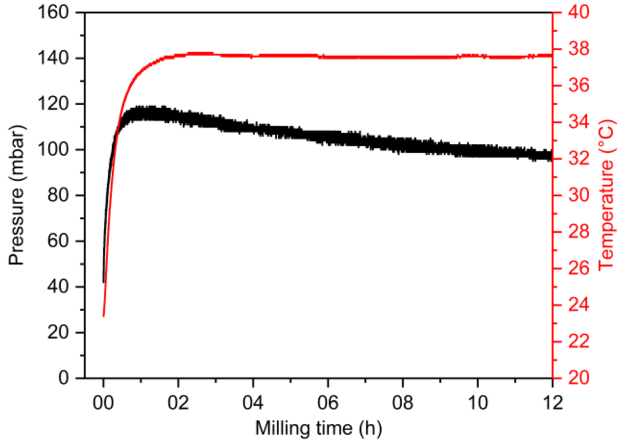


**Figure S11.** Pressure and temperature curves during the mechanochemical activation of $Mn_3O_4$ under argon atmosphere in a 45 mL grinding jar and 180 balls (diameter of 5 mm) made of $ZrO_2$ were used, the ball to powder ratio was 23:1 and with rotational speed of 400 rpm. The milling time was 12 hours.

Multi-fraction model in Rietveld refinement to describe the lithiation process of $Mn_3O_4$

As a possible structural model to describe the formation of the lithiation products, we used a multi-fraction model for each phase in order to achieve the best possible fit using the TOPAS 5 computer program. The data of the reference samples for $Mn_3O_4$ and $LiMn_3O_4$ provided for Figure S1 and Figure 4 were determined using a single fraction for the corresponding phases, however, other

samples also provide not only anisotropic line broadening, but also asymmetric reflections. This can be handled by fitting a single phase using a set of several fractions with similar lattice parameters and symmetric reflections. Therefore, if necessary, a second or even third fraction is implemented in the model for refinement of a single phase. In this case the values for *a* and *c* of the phase are calculated by a weighted averaging of the values of the fractions. The reference material $LiMn_3O_4$ also shows minor asymmetry of the peak shapes and a second fraction within the model provides an improvement ($R_{wp}$ = 7.10%).

From $Mn_3O_4$ to $LiMn_3O_4$ the lattice parameter *a* increases by about 29 pm while *c* decreases by about 49 pm. This is equivalent to $\Delta c = 1.67 \cdot \Delta a$. In addition, the volume increases by about 0.01471 $nm^3$ (4.68%).

The $Mn^{3+}$ ($d^4$) cations in $Mn_3O_4$ are distorted octahedrally coordinated and the oxygen atoms form a (distorted) cubic close packed lattice. The unit cell corresponds to a $\sqrt{2} \times \sqrt{2} \times 2$-fold superstructure of the Cu type. The structure of $LiMn_3O_4$ is similar to that of the tetragonal $Mn_3O_4$ and crystallizes also in the space group $I4_1/amd$.[3] Tetrahedrally coordinated manganese atoms are shifted to the nearest octahedrally coordinate site and the remaining octahedrally coordinated voids are filled with lithium atoms and the elementary cell is described by the $\sqrt{2} \times \sqrt{2} \times 2$-fold superstructure of the NaCl type.

Besides the educt $Mn_3O_4$ and the product $LiMn_3O_4$ some patterns reveal an additional transition state with the molecular formula $Li_{\sim 0.4}Mn_3O_4$ and flexibility of the lattice parameters of $Mn_3O_4$ and $LiMn_3O_4$. The lithium contents of those three phases were estimated from then volume of the unit cell assuming a linear dependency of the lithium contents *x* on volume. Therefore, a tetragonal model is proposed to describe the transition state from unlithiated $Mn_3O_4$ to lithiated $LiMn_3O_4$ (Figure S12). Here, the added lithium occupies the tetrahedral voids occupied by $Mn^{2+}$ at the Wyckoff position (WP) 4*a* or the adjacent, still free octahedral voids on WP 8*c*. This shift from WP 4*a* to WP 8*c* is also perceived by the manganese, although possibly to a different extent. For each structure as many boundary-constraints as possible are set, as far as not a higher flexibility was necessary. If more than one fraction was applied to fit a specific phase $Li_xMn_3O_4$ the mean volume $V_j$ was determined by weighted averaging the individual volumes $V_i$ (Equation 2). As weighting scheme, the mol fractions $n_i$ were implemented.

$$V_j = \frac{\sum_i n_i \cdot V_i}{\sum_i n_i} \tag{2}$$

The mol fractions $n_i$ were calculated from the weight fractions $wt_i$% and the lithium contents $x_i$. The values of $x_i$ were derived from the volumes $V_i$ assuming a linear dependency of the volume $V$ on $x$ (Equation 3), similar to the assumption for Vegards law.[4]

$$x_i = \frac{V_i - V_{0.0}}{V_{1.0} - V_{0.0}} \tag{3}$$

The reference values $V_{0.0}$ and $V_{1.0}$ were taken from the scans shown in Figure S1 and Figure 4. If the value $V_{1.0}$ is taken from literature[3, 5] instead of our reference scan, all values $x_i$ would increase by more than 19%. If an even larger volume $V$ of the $Li_xMn_3O_4$ phase were obtained by chemical lithiation, the $x_i$ values would have to be corrected to lower values as long as $x \leq 1$ can be assumed for this hypothetical reference sample. A chemical lithiation yielding $x > 1$ is not reliable, because lithium would be forced to occupy interstitial sites very close to other cation sites or a breakdown of the rock salt related structure would occur.

The lithium content of each sample is the product of the lithium content $x_j$ of each specific phase and its mole fraction $n_j$, the whole being summed over all phases (Equation 4). The mean value of the lithium content $<x>$ determined in this way is defined as follows:

$$< x > = \sum_j n_j \cdot x_j \tag{4}$$

The PXRD shows the change in powder diffractograms from the original starting material through slightly lithiated intermediates to the highly lithiated product phase. With the increase of the degree of lithiation, the reflections of $LiMn_3O_4$ become more dominant and with the simultaneous decrease of the reflex pattern of the unlithiated starting material. Figure S12 introduces a shorthand notation for the samples. The letter serves only as the name of the sample, after the first hyphen follows the duration of lithiation in hours and after the second hyphen the number of equivalents of the lithiation reagent. Thus, G-48-0.5 means that sample G was reacted for 48 hours with 0.5 equivalents of the lithiation reagent. The exact reaction conditions of the various samples are listed in Table S5.

The enlarged section demonstrates the evidence for the presence of a tetragonal transition state. The long, oblique lines show the reflections associated with the tetragonal transition state. These three reflexes change location and intensity as the degree of lithiation increases. In G-48-0.5 only

one shoulder can be observed and in the highly lithiated product the reflexes converge and form the two reflections (004) and (220). Figure S13 shows for even more samples the lithium contents and their phase fractions of the individual phases (non-lithiated to low lithiated, transition state, highly lithiated) in relation to the total content of the product obtained. It can also be seen how the transition state changes as lithiation progresses to the highly lithiated state.

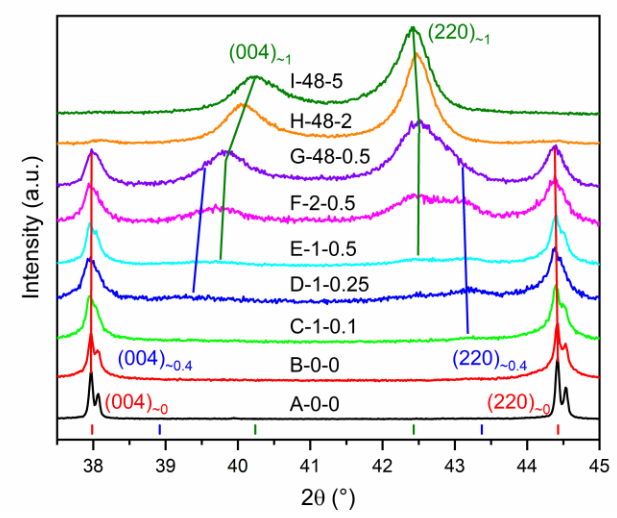


**Figure S12.** Enlarged section of PXRD patterns of $Li_xMn_3O_4$ with different grades of lithiation, which show evidence of the tetragonal transition state. Where the black font represents the reaction conditions in short form, here the letter is the name of the sample and after the first hyphen follows the lithiation time in hours and after the second hyphen follows the number of lithium equivalents used. The red lines indicate the *hkl*s of $Mn_3O_4$ the blue lines the *hkl*s of $Li_{0.4}Mn_3O_4$ and the green lines the *hkl*s of $LiMn_3O_4$. The long, oblique lines mark the tetragonal transition state.

**Table S5.** Milling time $t_{bm}$, rotational speed for ball milling *rs* time for lithiation $t_{Li}$ lithium equivalents $n_{Li}$ and reaction conditions *Rc* (stirring at room temperature (RT) or under reflux (rf)) of some samples of $Mn_3O_4$ and $Li_xMn_3O_4$.

| Sample | $t_{bm}$ (min) | *rs* (rpm) | $t_{Li}$ (h) | $n_{Li}$ (eq) | *Rc* (RT or rf) |
|---|---|---|---|---|---|
| (A) | 0 | 0 | 0 | 0 | - |
| (B) | 10 | 400 | 0 | 0 | - |
| (C) | 10 | 400 | 1 | 0.1 | RT |
| (D) | 10 | 400 | 1 | 0.25 | RT |
| (E) | 10 | 400 | 1 | 0.5 | RT |
| (F) | 10 | 400 | 2 | 0.5 | RT |
| (G) | 10 | 400 | 48 | 0.5 | rf |
| (H) | 10 | 200 | 48 | 2 | rf |
| (I) | 10 | 200 | 48 | 5 | rf |

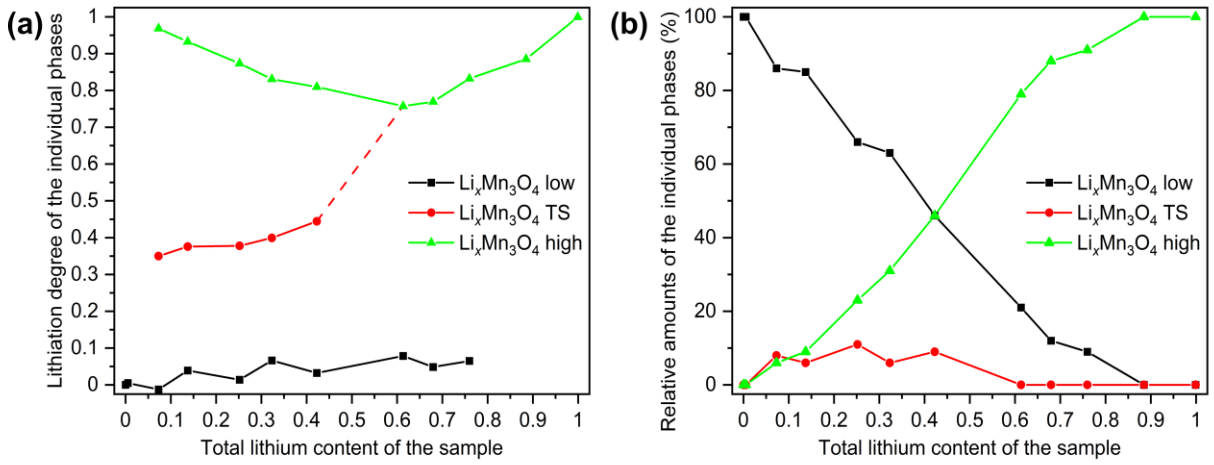


**Figure S13.** Lithiation degree of the individual phases (a) and relative amounts of the individual phases (b) vs total lithium content of the sample. Three phases with different lithium content were defined, $Li_xMn_3O_4$ low means non-lithiated to low lithiated $0 \leq x < 0.1$, $Li_xMn_3O_4$ TS means transition state $0.35 < x < 0.45$ and $Li_xMn_3O_4$ high means $0.7 < x \leq 1$.

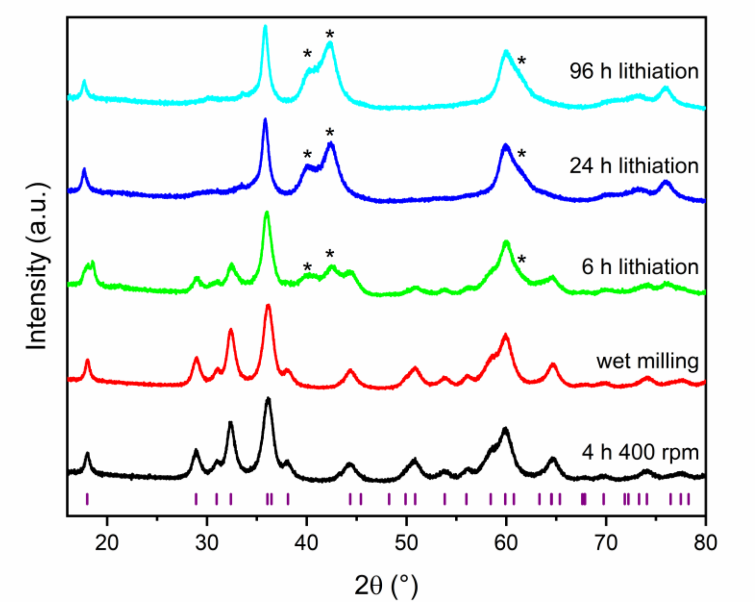


**Figure S14.** Enlarged section of the PXRD patterns of the starting materials and of the obtained products, when milled $Mn_3O_4$ (4 h, 400 rpm) was wet milled in *n*-pentane (30 min, 200 rpm, 180 milling balls consisting of yttrium stabilized zirconia and a diameter of 5 mm) and afterwards lithiated with *n*-butyllithium (2.5 equiva) for different time lengths (6 h, 24 h and 96 h) in *n*-hexane. The purple lines indicate the *hkl*s of $Mn_3O_4$. The asterisks mark the characteristic reflections of $LiMn_3O_4$.

**Table S6.** Average value of <$x$> in $Li_xMn_3O_4$ when milled $Mn_3O_4$ (4 h, 400 rpm) or the same substance wet milled in *n*-pentane is lithiated afterwards with *n*-butyllithium (2.5 equiv) in *n*-hexane for different time lengths.

| | *n*-butyllithium in *n*-hexane | |
|---|---|---|
| Time (h) | 4 h 400 rpm | Wet milled |
| 6 | 0.498 | 0.609 |
| 24 | 0.566 | 0.603 |
| 96 | 0.725 | 0.790 |

**Table S7.** Determined lithium contents via ICP-MS and Rietveld refinement, all samples were lithiated with 2.5 equiv of *n*-butyllithium except the first sample. This was lithiated twice with an excess of *n*-butyllithium (2 equiv and 5 equiv) to achieve maximum intercalation.

| Sample treatment | | | Lithium content $x$ in $Li_xMn_3O_4$ | |
|---|---|---|---|---|
| Milling time (min) | Rotational speed (rpm) | Lithiation time (h) | ICP-MS | Rietveld-Refinement |
| 10 | 200 | 48 | 1.321 | 1.000 |
| 10 | 400 | 3 | 0.621 | 0.490 |
| 10 | 600 | 3 | 0.490 | 0.380 |
| 10 | 200 | 96 | 0.736 | 0.556 |
| 10 | 400 | 96 | 1.236 | 0.862 |

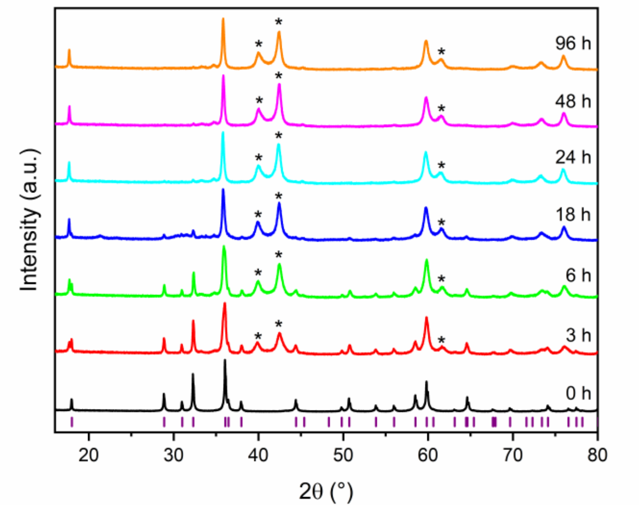


**Figure S15.** Enlarged section of the PXRD patterns of the obtained products, when milled $Mn_3O_4$ (10 min, 400 rpm, 180 milling balls consisting of yttrium stabilized zirconia and a diameter of 5 mm) was lithiated with *n*-butyllithium (2.5 equiv) in *n*-hexane for different time lengths. The purple lines indicate the *hkl*s of $Mn_3O_4$. The asterisks mark the characteristic reflections of $LiMn_3O_4$.

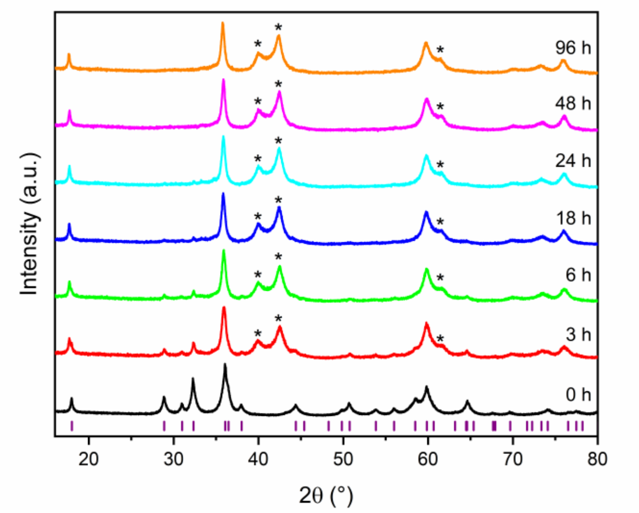


**Figure S16.** Enlarged section of the PXRD patterns of the obtained products, when milled $Mn_3O_4$ (1 h, 400 rpm, 180 milling balls consisting of yttrium stabilized zirconia and a diameter of 5 mm) was lithiated with *n*-butyllithium (2.5 equiv) in *n*-hexane for different time lengths. The purple lines indicate the *hkl*s of $Mn_3O_4$. The asterisks mark the characteristic reflections of $LiMn_3O_4$.

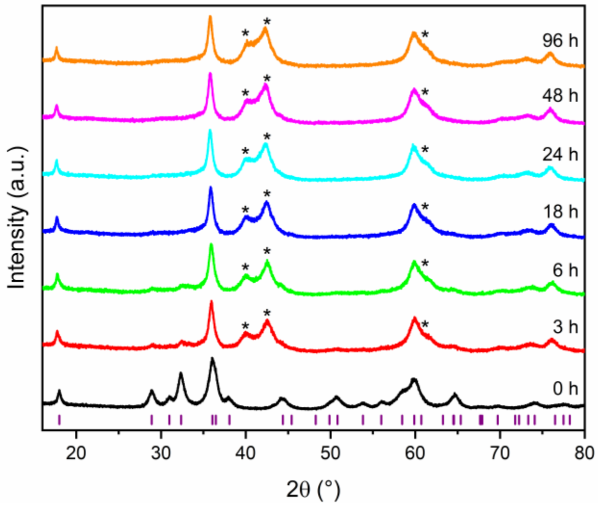


**Figure S17.** Enlarged section of the PXRD patterns of the obtained products, when milled $Mn_3O_4$ (2 h, 400 rpm, 180 milling balls consisting of yttrium stabilized zirconia and a diameter of 5 mm) was lithiated with *n*-butyllithium (2.5 equiv) in *n*-hexane for different time lengths. The purple lines indicate the *hkl*s of $Mn_3O_4$. The asterisks mark the characteristic reflections of $LiMn_3O_4$.

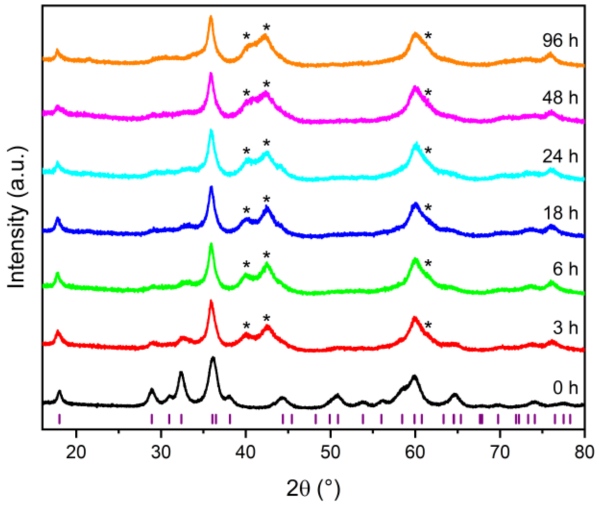


**Figure S18.** Enlarged section of the PXRD patterns of the obtained products, when milled $Mn_3O_4$ (4 h, 400 rpm, 180 milling balls consisting of yttrium stabilized zirconia and a diameter of 5 mm) was lithiated with *n*-butyllithium (2.5 equiv) in *n*-hexane for different time lengths. The purple lines indicate the *hkl*s of $Mn_3O_4$. The asterisks mark the characteristic reflections of $LiMn_3O_4$.

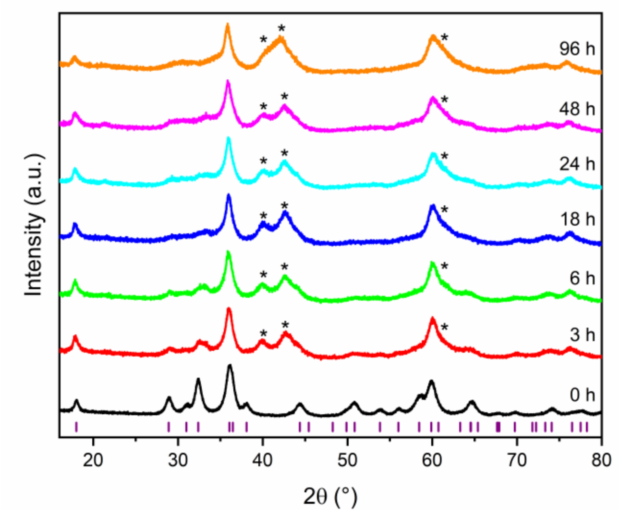


**Figure S19.** Enlarged section of the PXRD patterns of the obtained products, when milled $Mn_3O_4$ (8 h, 400 rpm, 180 milling balls consisting of yttrium stabilized zirconia and a diameter of 5 mm) was lithiated with *n*-butyllithium (2.5 equiv) in *n*-hexane for different time lengths. The purple lines indicate the *hkl*s of $Mn_3O_4$. The asterisks mark the characteristic reflections of $LiMn_3O_4$.

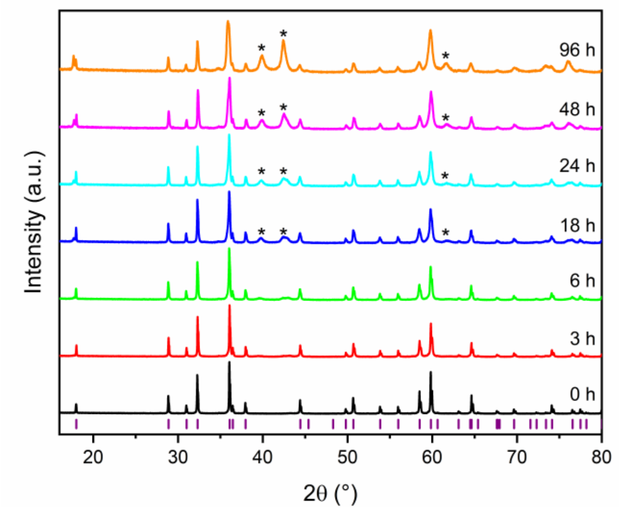


**Figure S20.** Enlarged section of the PXRD patterns of the obtained products, when milled $Mn_3O_4$ (10 min, 200 rpm, 180 milling balls consisting of yttrium stabilized zirconia and a diameter of 5 mm) was lithiated with *n*-butyllithium (2.5 equiv) in *n*-hexane for different time lengths. The purple lines indicate the *hkl*s of $Mn_3O_4$. The asterisks mark the characteristic reflections of $LiMn_3O_4$.

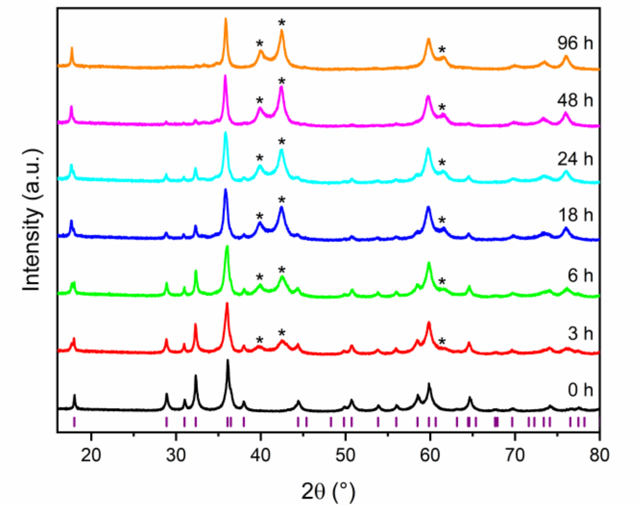


**Figure S21.** Enlarged section of the PXRD patterns of the obtained products, when milled $Mn_3O_4$ (10 min, 600 rpm, 180 milling balls consisting of yttrium stabilized zirconia and a diameter of 5 mm) was lithiated with *n*-butyllithium (2.5 equiv) in *n*-hexane for different time lengths. The purple lines indicate the *hkl*s of $Mn_3O_4$. The asterisks mark the characteristic reflections of $LiMn_3O_4$.

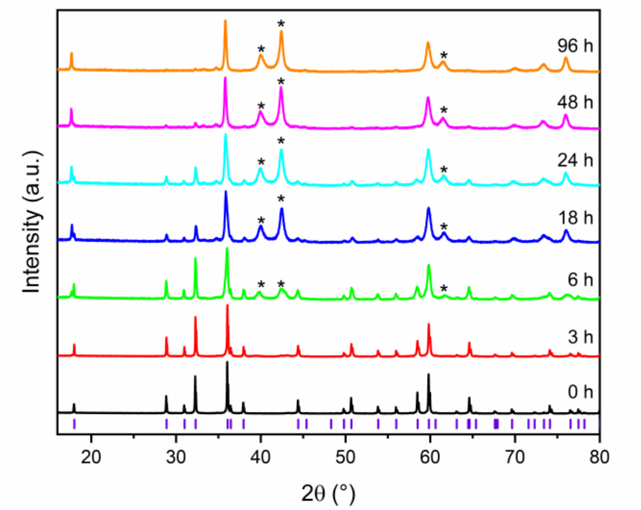


**Figure S22.** Enlarged section of the PXRD patterns of the obtained products, when milled $Mn_3O_4$ (30 min, 200 rpm, 180 milling balls consisting of yttrium stabilized zirconia and a diameter of 5 mm) was lithiated with *n*-butyllithium (2.5 equiv) in *n*-hexane for different time lengths. The purple lines indicate the *hkl*s of $Mn_3O_4$. The asterisks mark the characteristic reflections of $LiMn_3O_4$.

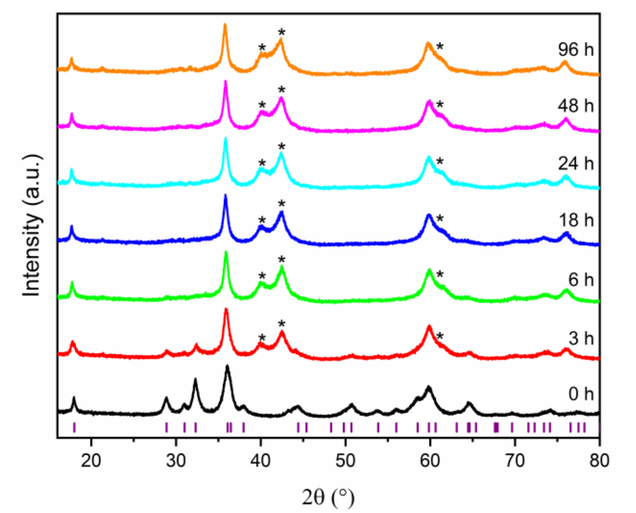


**Figure S23.** Enlarged section of the PXRD patterns of the obtained products, when milled $Mn_3O_4$ (30 min, 600 rpm, 180 milling balls consisting of yttrium stabilized zirconia and a diameter of 5 mm) was lithiated with *n*-butyllithium (2.5 equiv) in *n*-hexane for different time lengths. The purple lines indicate the *hkl*s of $Mn_3O_4$. The asterisks mark the characteristic reflections of $LiMn_3O_4$.

**Table S8.** Aggregation numbers of organolithium reagents depending on the solvent used.[6]

| Organolithium reagent | Solvent | Aggregation number |
|---|---|---|
| *n*-butyllithium | *n*-hexane | 6 |
| | diethyl ether | 4 |
| | THF | 4/2 |
| *tert*-butyllithium | *n*-hexane | 4 |
| methyllithium | diethyl ether | 4 |
| | THF | 4 |
| *n*-hexyllithium | *n*-hexane | no data |

**Table S9.** Mean value of <$x$> in $Li_xMn_3O_4$ when using different lithiation agents for lithiation (*tert*-butyllithium, *n*-butyllithium or methyllithium) of pristine $Mn_3O_4$ depending on the different solvents used.

| | *n*-hexane | | THF | | diethyl ether | |
|---|---|---|---|---|---|---|
| Time (h) | *tert*-BuLi | *n*-BuLi | *n*-BuLi | MeLi | *n*-BuLi | MeLi |
| 3 | 0.082 | 0.182 | 0.146 | 0.094 | 0.228 | 0.111 |
| 6 | 0.123 | 0.252 | 0.140 | 0.124 | 0.267 | 0.122 |
| 18 | 0.188 | 0.426 | 0.159 | 0.150 | 0.445 | 0.178 |
| 24 | 0.236 | 0.501 | 0.134 | 0.168 | 0.646 | 0.204 |
| 48 | 0.189 | 0.667 | 0.139 | 0.241 | 0.779 | 0.285 |
| 96 | 0.261 | 0.705 | 0.154 | 0.291 | 0.882 | 0.346 |

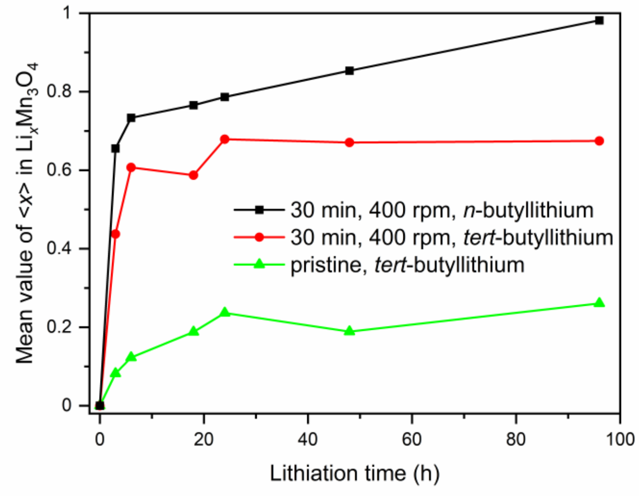


**Figure S24.** Mean value <$x$> of $Li_xMn_3O_4$ depending on different lithiation reagents and time of lithiation. The lithiation of pristine or mechanochemically activated $Mn_3O_4$ (30 min, 400 rpm) was performed with 2.5 equiv *tert*-butyllithium in *n*-hexane. For comparison, the determined value of $Li_xMn_3O_4$ during lithiation of mechanochemically activated $Mn_3O_4$ (30 min, 400 rpm) with 2.5 equiv *n*-butyllithium in *n*-hexane is also shown. The amount of $Li_xMn_3O_4$ was determined by Rietveld refinement.

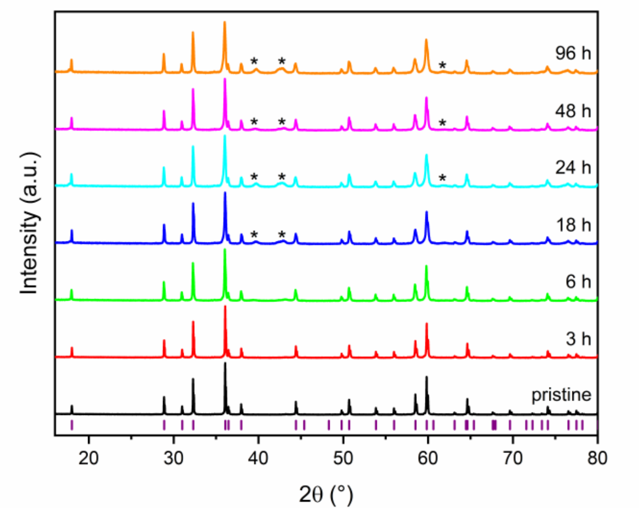


**Figure S25.** Enlarged section of the PXRD patterns of the obtained products, when unmilled $Mn_3O_4$ was lithiated with *tert*-butyllithium (2.5 equiv) in *n*-hexane for different time lengths. The purple lines indicate the *hkl*s of $Mn_3O_4$. The asterisks mark the characteristic reflections of $LiMn_3O_4$.

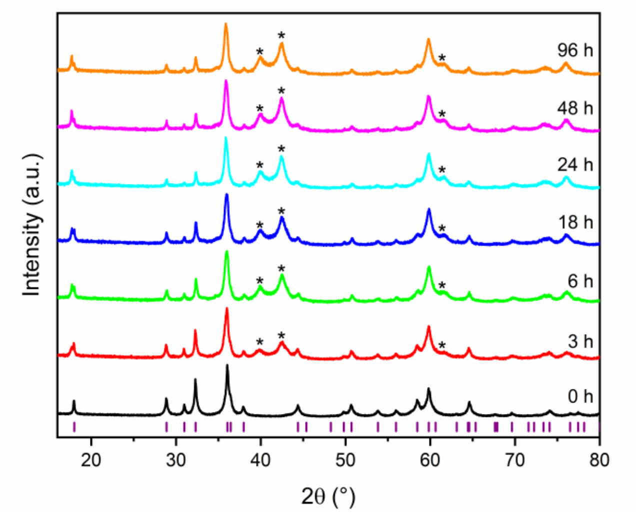


**Figure S26.** Enlarged section of the PXRD patterns of the obtained products, when milled $Mn_3O_4$ (30 min, 400 rpm, 180 milling balls consisting of yttrium stabilized zirconia and a diameter of 5 mm) was lithiated with *tert*-butyllithium (2.5 equiv) in *n*-hexane for different time lengths. The purple lines indicate the *hkl*s of $Mn_3O_4$. The asterisks mark the characteristic reflections of $LiMn_3O_4$.

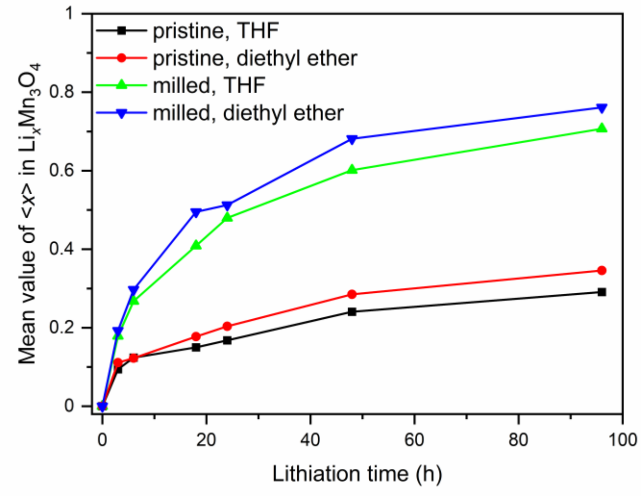


**Figure S27.** Mean value <*x*> of $Li_xMn_3O_4$ depending on the solvent used (THF or diethyl ether) and time of lithiation. The lithiation of pristine or mechanochemically activated $Mn_3O_4$ (30 min, 400 rpm) was performed with 2.5 equiv methyllithium. The amount of $Li_xMn_3O_4$ was determined by Rietveld refinement.

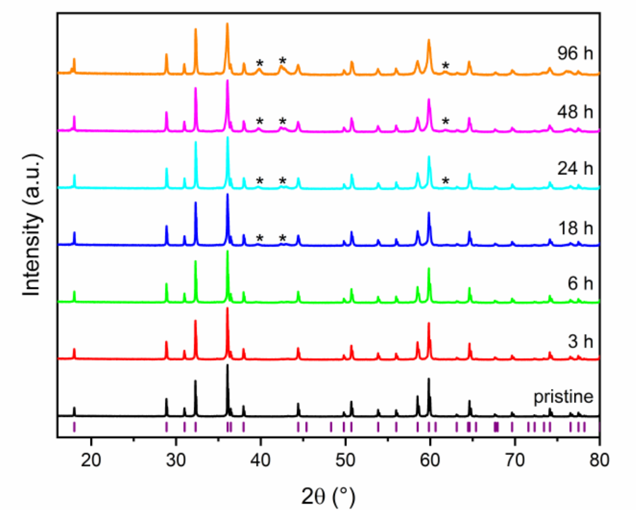


**Figure S28.** Enlarged section of the PXRD patterns of the obtained products, when unmilled $Mn_3O_4$ was lithiated with methyllithium (2.5 equiv) in THF for different time lengths. The purple lines indicate the *hkl*s of $Mn_3O_4$. The asterisks mark the characteristic reflections of $LiMn_3O_4$.

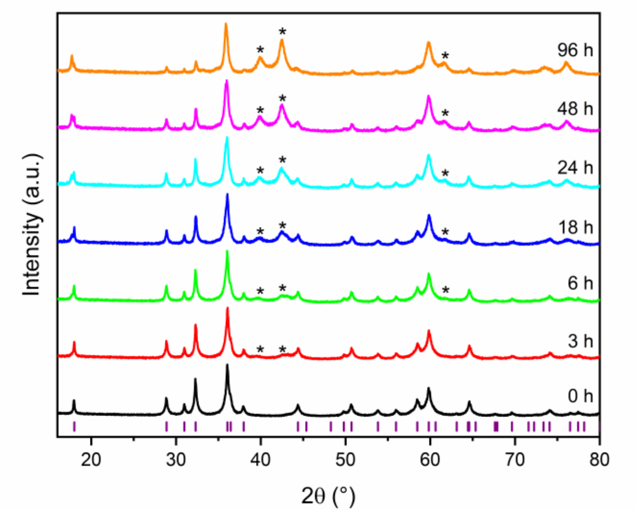


**Figure S29.** Enlarged section of the PXRD patterns of the obtained products, when milled $Mn_3O_4$ (30 min, 400 rpm, 180 milling balls consisting of yttrium stabilized zirconia and a diameter of 5 mm) was lithiated with methyllithium (2.5 equiv) in THF for different time lengths. The purple lines indicate the *hkl*s of $Mn_3O_4$. The asterisks mark the characteristic reflections of $LiMn_3O_4$.

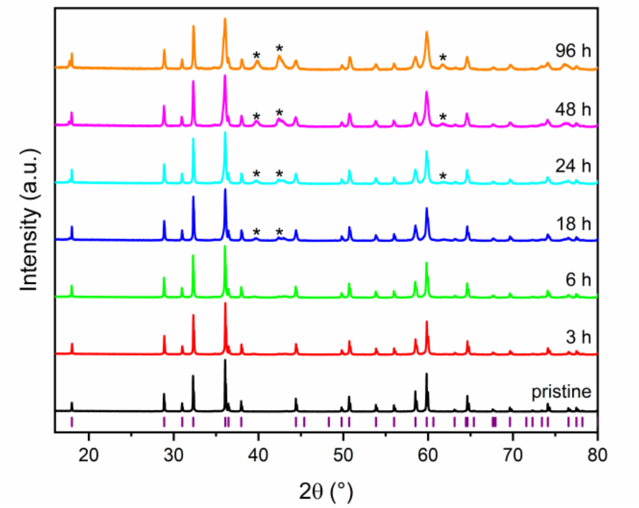


**Figure S30.** Enlarged section of the PXRD patterns of the obtained products, when unmilled $Mn_3O_4$ was lithiated with methyllithium (2.5 equiv) in diethyl ether for different time lengths. The purple lines indicate the *hkl*s of $Mn_3O_4$. The asterisks mark the characteristic reflections of $LiMn_3O_4$.

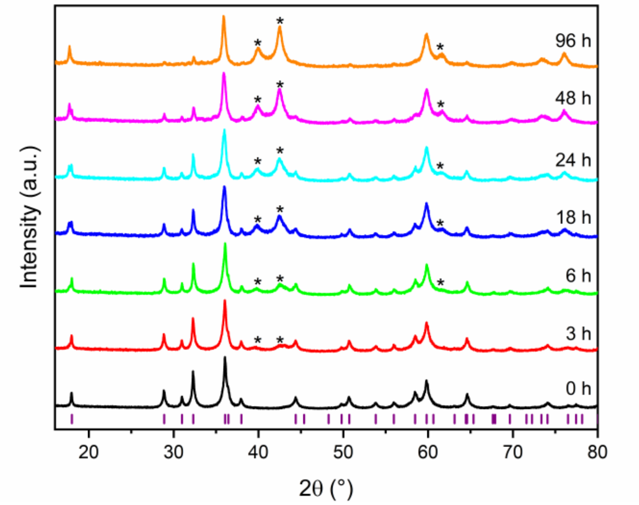


**Figure S31.** Enlarged section of the PXRD patterns of the obtained products, when milled $Mn_3O_4$ (30 min, 400 rpm, 180 milling balls consisting of yttrium stabilized zirconia and a diameter of 5 mm) was lithiated with methyllithium (2.5 equiv) in diethyl ether for different time lengths. The purple lines indicate the *hkl*s of $Mn_3O_4$. The asterisks mark the characteristic reflections of $LiMn_3O_4$.

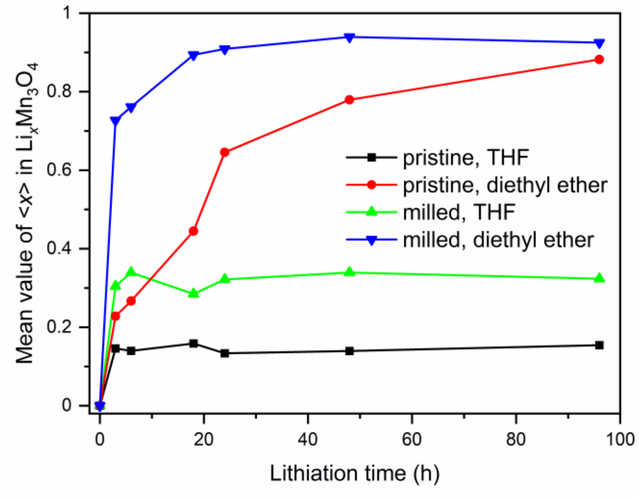


**Figure S32.** Mean value <$x$> of $Li_xMn_3O_4$ depending on the solvent used (THF or diethyl ether) and time of lithiation. The lithiation of pristine or mechanochemically activated $Mn_3O_4$ (30 min, 400 rpm) was performed with 2.5 equiv *n*-butyllithium. The amount of $Li_xMn_3O_4$ was determined by Rietveld refinement.

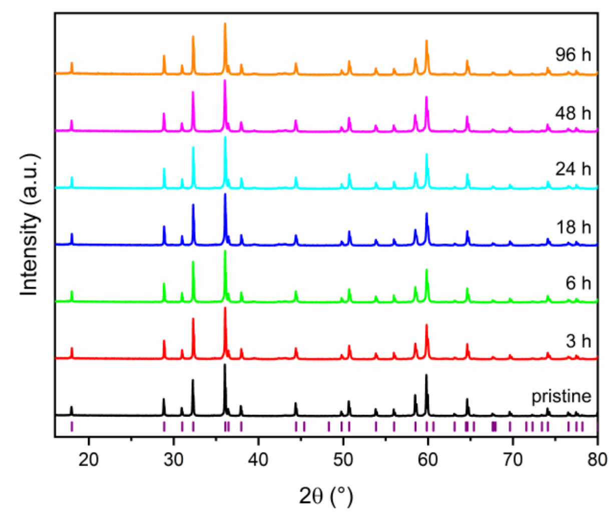


**Figure S33.** Enlarged section of the PXRD patterns of the obtained products, when unmilled $Mn_3O_4$ was lithiated with *n*-butyllithium (2.5 equiv) in THF for different time lengths. The purple lines indicate the *hkl*s of $Mn_3O_4$.

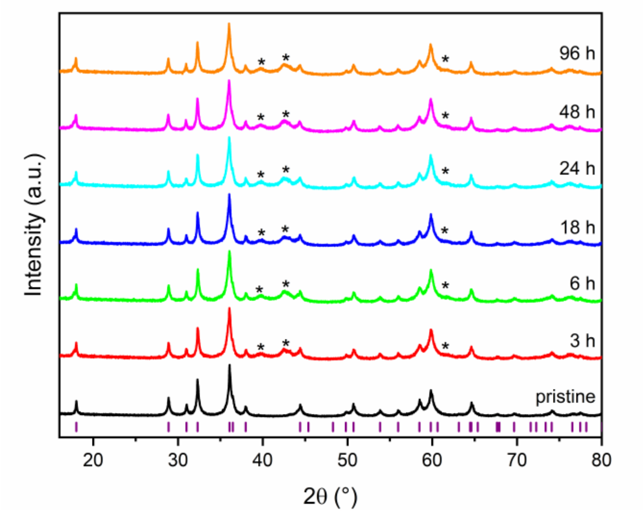


**Figure S34.** Enlarged section of the PXRD patterns of the obtained products, when milled $Mn_3O_4$ (30 min, 400 rpm, 180 milling balls consisting of yttrium stabilized zirconia and a diameter of 5 mm) was lithiated with *n*-butyllithium (2.5 equiv) in THF for different time lengths. The purple lines indicate the *hkl*s of $Mn_3O_4$. The asterisks mark the characteristic reflections of $LiMn_3O_4$.

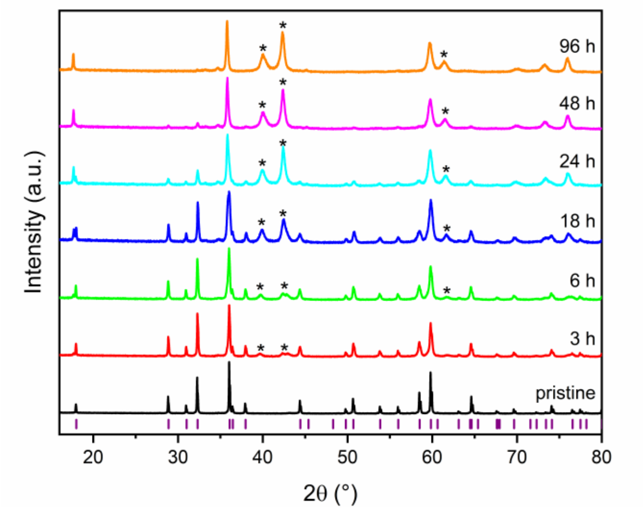


**Figure S35.** Enlarged section of the PXRD patterns of the obtained products, when unmilled $Mn_3O_4$ was lithiated with *n*-butyllithium (2.5 equiv) in diethyl ether for different time lengths. The purple lines indicate the *hkl*s of $Mn_3O_4$. The asterisks mark the characteristic reflections of $LiMn_3O_4$.

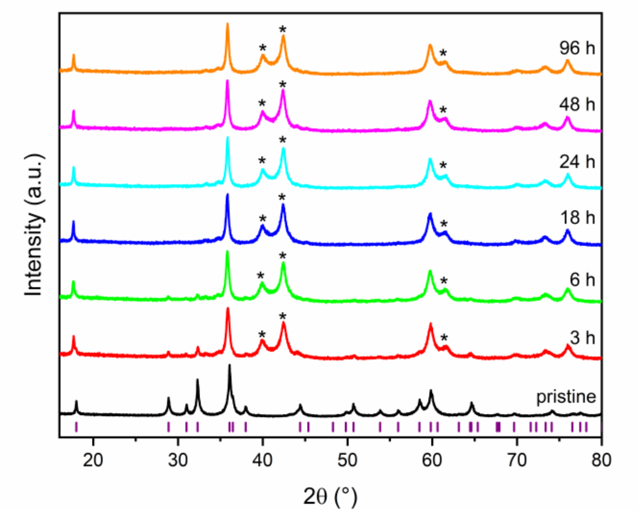


**Figure S36.** Enlarged section of the PXRD patterns of the obtained products, when milled $Mn_3O_4$ (30 min, 400 rpm, 180 milling balls consisting of yttrium stabilized zirconia and a diameter of 5 mm) was lithiated with *n*-butyllithium (2.5 equiv) in diethyl ether for different time lengths. The purple lines indicate the *hkl*s of $Mn_3O_4$. The asterisks mark the characteristic reflections of $LiMn_3O_4$.

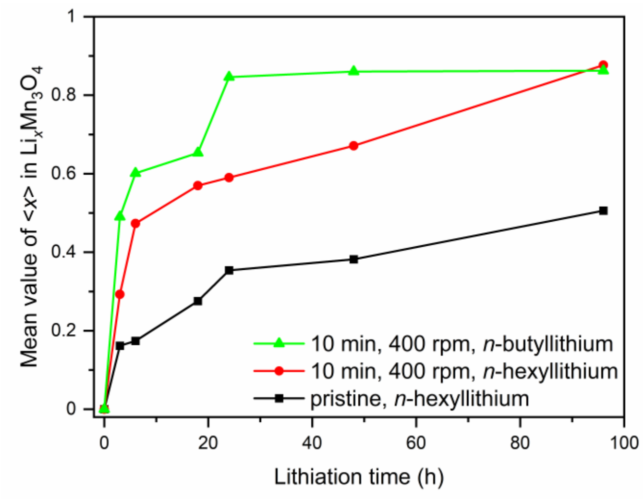


**Figure S37.** Mean value <*x*> of $Li_xMn_3O_4$ depending on different lithiation reagents and time of lithiation. The lithiation of pristine or mechanochemically activated $Mn_3O_4$ (10 min, 400 rpm) was performed with 2.5 equiv *n*-hexyllithium in *n*-hexane. For comparison, the determined value of $Li_xMn_3O_4$ during lithiation of mechanochemically activated $Mn_3O_4$ (10 min, 400 rpm) with 2.5 equiv *n*-butyllithium in *n*-hexane is also shown. The amount of $Li_xMn_3O_4$ was determined by Rietveld refinement.

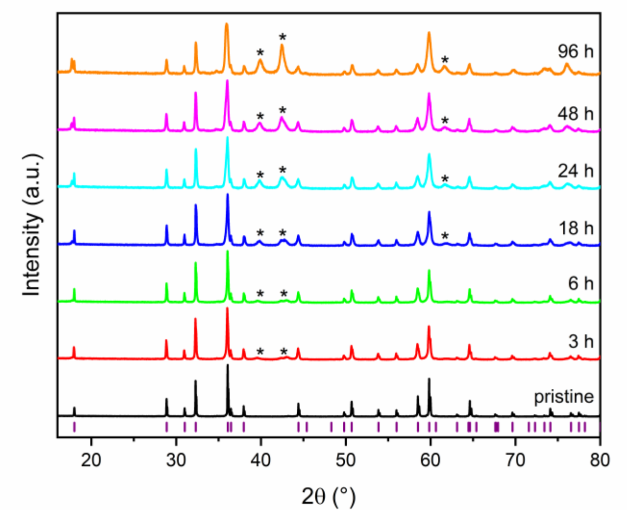


**Figure S38.** Enlarged section of the PXRD patterns of the obtained products, when unmilled $Mn_3O_4$ was lithiated with *n*-hexyllithium (2.5 equiv) in *n*-hexane for different time lengths. The purple lines indicate the *hkl*s of $Mn_3O_4$. The asterisks mark the characteristic reflections of $LiMn_3O_4$.

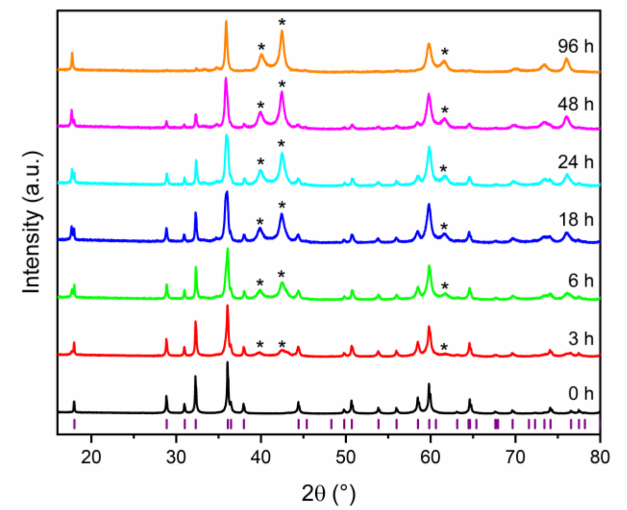


**Figure S39.** Enlarged section of the PXRD patterns of the obtained products, when milled $Mn_3O_4$ (10 min, 400 rpm, 180 milling balls consisting of yttrium stabilized zirconia and a diameter of 5 mm) was lithiated with *n*-hexyllithium (2.5 equiv) in *n*-hexane for different time lengths. The purple lines indicate the *hkl*s of $Mn_3O_4$. The asterisks mark the characteristic reflections of $LiMn_3O_4$.

## Results of the lithiation of $Mn_3O_4$ with lithium sulfide

After filtration under argon and acetonitrile washing, the sample was dried and prepared in a glovebox for measurement in an airtight dome sample carrier, excluding oxygen and moisture. No lithium intercalation was observed initially. Upon opening the dome and exposing the sample to ambient air, changes in the PXRD pattern were observed: $Li_2S$ remained visible when the dome was opened briefly, after 2 days of air exposure, lithium hydroxide (LiOH) formation was detected. Prolonged air exposure led to further conversion to lithium carbonate ($Li_2CO_3$). The PXRD data indicates that air exposure causes gradual transformation of the initial $Li_2S$ phase to lithium hydroxide and then lithium carbonate.

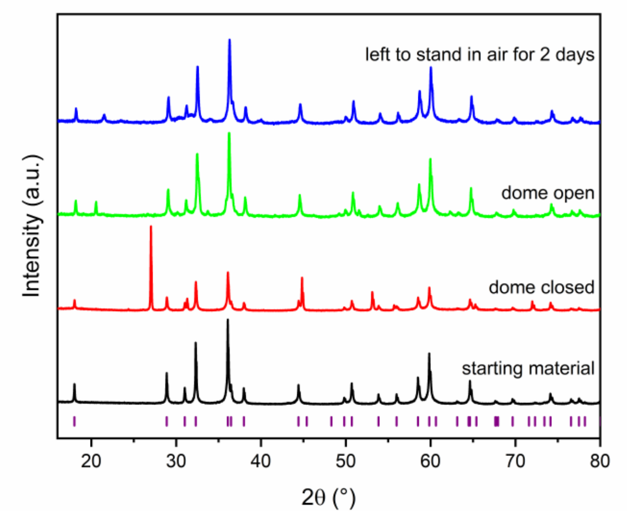


**Figure S40.** Enlarged section of the PXRD patterns of the obtained products, when milled $Mn_3O_4$ (10 min, 400 rpm, 180 milling balls consisting of yttrium stabilized zirconia and a diameter of 5 mm) was lithiated with lithium sulfide (5 equiv) in acetonitrile for 4 days at room temperature. The sample was measured under different conditions, to illustrate what happens to the sample, when it gets in contact with oxygen and/or humidity. The purple lines indicate the *hkl*s of $Mn_3O_4$.

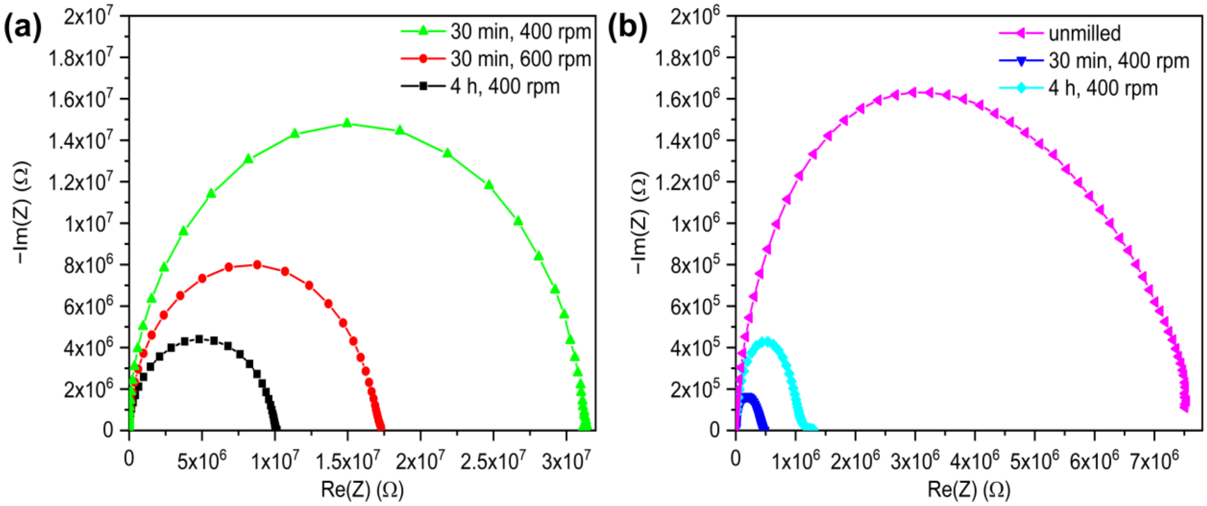


**Figure S41.** Nyquist plots of $Mn_3O_4$ samples (a) mechanochemically activated under different conditions and of unmilled and milled $Li_xMn_3O_4$ samples (b).

## References


(1) McMurdie, H. F.; Sullivan, B. M.; Mauer, F. A., High-temperature X-ray study of the system $Fe_3O_4$-$Mn_3O_4$. *J. Res. Nat. Bur. Stand.* **1950,** *45*, 35-41.

(2) Deljoo, B.; Tan, H.; Suib, S. L.; Aindow, M., Thermally activated structural transformations in manganese oxide nanoparticles under air and argon atmospheres. *J. Mater. Sci.* **2020,** *55*, 7247-7258.

(3) Goodenough, J. B.; Thackeray, M. M.; David, W. I. F.; Bruce, P. G., Lithium insertion/extraction reactions with manganese oxides. *Rev. Chim. Miner.* **1984,** *21*, 435-455.

(4) Vegard, L., Die Konstitution der Mischkristalle und die Raumfüllung der Atome. *Z. Phys.* **1921,** *5*, 17-26.

(5) Lowe, M. A.; Gao, J.; Abruña, H. D., *In operando* X-ray studies of the conversion reaction in $Mn_3O_4$ lithium battery anodes. *J. Mater. Chem. A* **2013,** *1*, 2094-2103.

(6) Rathman, T. L.; Schwindeman, J. A., Preparation, Properties, and Safe Handling of Commercial Organolithiums: Alkyllithiums, Lithium *sec*-Organoamides, and Lithium Alkoxides. *Org. Process Res. Dev.* **2014,** *18*, 1192-1210.
